# Structural Motifs in Soft Fibrous Tissues: Revealing Structure‐Mechanics Relationships in Deformation and Tear Resistance for Biomimetic Material Design

**DOI:** 10.1002/adhm.202500153

**Published:** 2025-07-07

**Authors:** Mirit Sharabi

**Affiliations:** ^1^ Department of Mechanical Engineering and Mechatronics Ariel University Ariel 407000 Israel

**Keywords:** biomimetics, mechanical behavior, soft fibrous tissues, structural motifs, structure‐function relationship

## Abstract

Over millions of years, nature has optimized soft fibrous tissues (SFTs) to exhibit exceptional mechanical properties—characteristics that remain challenging to replicate in engineered materials. These properties emerge from simple, repeating building blocks organized into complex structural motifs, which collectively enable diverse and robust mechanical functions. Although such motifs are common across SFTs, their specific composition and architecture define the physiological roles of each tissue. There is a growing need to develop tissue constructs and implantable devices that replicate the structural integrity, mechanical performance, and long‐term functionality of native tissues across multiple hierarchical levels. Despite the clinical relevance of mimicking SFT mechanics in synthetic systems, the fundamental structure–mechanics relationships underpinning these behaviors remain incompletely understood. This review synthesizes recent advances in elucidating how structural motifs in SFTs contribute to their mechanical performance and explores how these principles can inform the design of biomimetic materials. It further highlights emerging strategies in soft‐engineered material systems and their mechanical characterization. By clarifying the links between tissue architecture and properties such as deformation and tear resistance, this work proposes design guidelines for developing the next generation of resilient, functional, and translationally relevant bioengineered materials.

## Graphical abstract

This review explores how soft fibrous tissues achieve their remarkable mechanical behavior through recurring structural motifs across multiple length scales. It highlights key advances in mimicking these features using fiber‐reinforced hydrogels and hierarchical composites, offering insights into mechanical compatibility, flaw tolerance, and tear resistance essential for designing next‐generation biomimetic materials and soft‐tissue replacements.

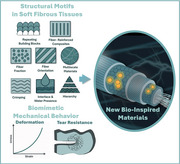

## 1 Introduction

Over millions of years, nature has refined the design of soft fibrous tissues (SFTs) to achieve remarkable mechanical properties that engineered materials have yet to replicate fully. The mechanical behavior of SFTs offers an optimal combination of stiffness (resistance to elastic deformation), strength (resistance to plastic deformation), and toughness (resistance to fracture)—properties essential for performance under demanding, long‐term physiological conditions. Where most synthetic materials exhibit either high strength or toughness, but rarely both, nature has resolved the strength‐toughness conflict using unique structural motifs, enabling the multifunctionality and multiple exceptional mechanical behaviors such as strain stiffening, large deformations, and unparalleled tear‐resistance.^[^ ^1^ ^]^

While hard biological materials such as nacre and bone have received considerable attention for their exceptional properties and well‐studied structure‐mechanics relationships,^[^ ^2^, ^3^, ^4^ ^]^ soft biological materials (such as SFTs) remain underexplored. Unveiling the structural motifs that govern SFT mechanics requires an understanding of how nature leverages hierarchical design to optimize function. These principles can then be translated into biomaterial design to bridge hierarchical scales to achieve integrated tissue functionality.

This review aims to define structural motifs in SFTs and examine their contributions to mechanical behavior. These insights are intended to inform the development of synthetic tissue constructs and implantable materials that exhibit structural integrity, mechanical biocompatibility,^[^ ^5^ ^]^ and sustainable functionality across hierarchical levels. By identifying recurring motifs in SFTs and assessing their functional roles, we propose design strategies for bioengineered materials that closely mimic the performance of native tissues.

Although biological compatibility remains a foundational requirement, mechanical biocompatibility^[^ ^5^ ^]^—the ability of a material to mimic the load‐bearing, deformation, and failure characteristics of native tissue—is increasingly recognized as a prerequisite for long‐term success. While biological compatibility governs immune response and cellular integration, mechanical compatibility determines load transfer, tissue integration, and structural durability, particularly in SFTs subjected to complex, multiaxial loading.

Furthermore, we focus on key phenomena observed in specific SFTs and the application of an iterative reverse biomimetic approach^[^ ^2^, ^6^, ^7^, ^8^ ^]^ through simplified material systems. By isolating these effects, we aim to deepen the understanding of structure‐function relationships and elucidate the distinct mechanisms involved. This dual approach offers reciprocal benefits: engineered materials can reveal hidden structural motifs and uncover previously unknown biomechanical mechanisms in biological tissues while inspiring the design of advanced biomimetic materials.

Structurally, all SFTs function as composite materials comprising three primary building blocks: collagen, elastin, and proteoglycans (PGs) or glycosaminoglycans (GAGs). While these components contribute to both biological and mechanical functions, this review emphasizes their mechanical roles as reinforcing, elastic, and viscoelastic elements, respectively.

As in classical fiber‐reinforced composites, the fiber volume fraction and the orientation of collagen fibers have an essential role in determining the mechanical behavior of SFTs. However, these parameters alone cannot explain the breadth of their mechanical capabilities. Indeed, additional structural motifs, including multiscale micro‐nano fiber structures, crimping, and weak interfaces, contribute to the intricate structural complexity of SFTs and their resulting mechanics,^[^ ^9^ ^]^ including improved fracture and fatigue resistance.

Incorporating these structural motifs into engineered materials is vital for achieving the mechanical biocompatibility and durability of native tissues. These insights hold promise not only for biomedical applications but also for emerging fields such as soft robotics, aerospace, and advanced wearables, where multifunctional, damage‐resistant soft materials are increasingly needed.

## 2 Structural Motifs

**Figure 1:**
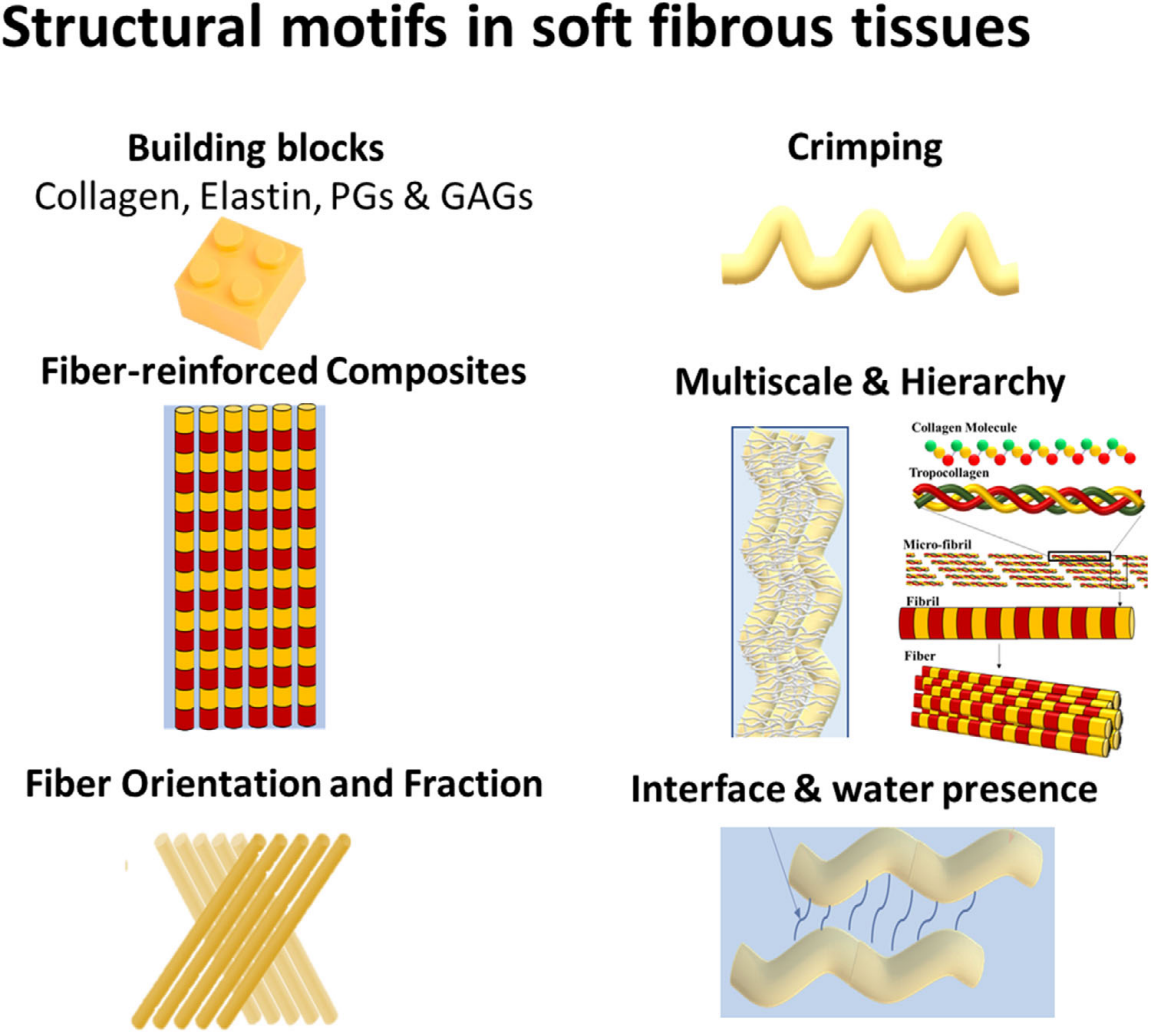
Schematic illustration of key structural motifs in soft fibrous tissues, including: repeating building blocks; fiber‐reinforced composite architecture; fiber volume fraction; fiber orientation and orientation distribution; fiber crimping; interfacial features and water content; hierarchical organization; and multiscale structural integration.

Soft fibrous tissues are characterized by several repeating motifs that govern their mechanical behavior: Repeating building blocks, Fiber‐reinforced composites, Fiber fraction, Fiber Orientation (and orientation distribution), Crimping, Interface and water presence, Hierarchy and Multiscale materials (**Figure** **1**). While these motifs are the most common, additional motifs like superhydrophilicity, gradients, and radial connectivity are also present in various SFTs. These motifs enable unparalleled large deformation behavior, characteristic strain stiffening behavior, exceptional fracture toughness, and crack shielding capabilities.

### 2.1 Building Blocks and Fiber‐Reinforced Composites

From a materials science perspective, all SFTs can be viewed as composite materials composed of three recurring building blocks: *Collagen, Elastin*, and *PGs*, each serving a distinct mechanical function.^[^ ^9^ ^]^ Although SFTs also include additional components that influence mechanical performance and interactions between building blocks, this review simplifies these complexities to establish broadly applicable design principles for engineered materials.

Structurally, collagen fibers act as the reinforcing agent, whereas the matrix comprises the remaining building blocks. *Collagen* (fibrous, type I), often referred to as “the steel of biological materials,”^[^ ^11^ ^]^ serves as the primary load‐bearing element, providing stiffness and strength. Its unique hierarchical structure is responsible for the characteristic nonlinear J‐shaped mechanical behavior of tissues, as extensively reviewed.^[^ ^4^, ^9^, ^10^, ^11^, ^12^ ^]^

*Elastin and associated elastic fibers* provide flexibility and stretchability, similar to elastomers. Typically found in a complex with microfibrils, elastin enables high strain tolerance (up to 70% strain), linear elastic behavior, and fatigue resistance with minimal hysteresis loss under tension.^[^ ^13^, ^14^, ^15^, ^16^ ^]^ It primarily allows elastic recoil in soft tissues, similar to natural rubber. This function is hydration‐dependent: elastin becomes flexible when hydrated, with PGs and GAGs contributing to tissue hydration and impact absorption.^[^ ^17^, ^18^ ^]^

*PGs* and *GAGs* are negatively charged and absorb water up to 1000 times their volume, providing tissues with an aqueous environment, viscoelasticity, and shock‐absorption ability.^[^ ^9^, ^10^, ^19^, ^20^ ^]^ In tendons, they are located between collagen fibrils, binding them noncovalently,^[^ ^21^ ^]^ which enables load transfer, sliding, and stretching of the fibrils.^[^ ^22^ ^]^ PGs and GAGs are present at all hierarchical levels of the tissue and have been identified down to the fibril scale.^[^ ^9^ ^]^

Different SFTs consist of distinct combinations of building blocks and structural motifs, resulting in a wide range of mechanical properties.

Substantial variability exists across tissue types, species, and anatomical regions. This variability is further compounded by differences in testing protocols, mechanical definitions, sample orientation, and processing methods. The lack of standardized data and methodologies complicates direct comparison. Moreover, general guidelines for systematically studying the structure–mechanics relationships in SFTs remain limited. Representative values are provided in **Table** **1** to illustrate typical structural and mechanical properties (deformation behavior) based on the literature.^[^ ^10^, ^16^, ^17^, ^23^, ^24^, ^25^, ^26^, ^27^, ^28^, ^29^, ^30^, ^31^, ^32^, ^33^, ^34^, ^35^, ^36^, ^37^, ^38^, ^39^, ^40^, ^41^, ^42^, ^43^, ^44^, ^45^, ^46^, ^47^, ^48^ ^]^ Fracture properties were excluded from the table due to the extreme variability in testing techniques and the absence of standardized definitions, which currently preclude meaningful comparison. As a result, this review emphasizes qualitative descriptions of damage tolerance mechanisms rather than absolute fracture values.

**Table 1:** Representative structural and mechanical properties of different SFTs.

Tissue	Building Blocks^c)^			Structural Motifs^a)^, ^b)^				Mechanical Properties^a)^, ^b)^		
	Collagen [%]	Elastin [%]	PGs & GAGs [%]	Fibril Diameter [nm]	Main Orientation	Crimp Morphology [Angle]	Crimp Period [µm]	Young's Modulus^d)^ [MPa]	UTS [MPa]	Ultimate Strain [%]
Tendon	75–85	<3	1–5	40‐340	Uniaxial	Zigzag (6–19°)	40–284	1000–2000	50–140	10–15
Ligament	70–80	10–15	4–7	40‐300	Uniaxial	Zigzag (25°)	∼32	200–400	50–100	10–15
Annulus Fibrosus	59–67	2	7–9	20‐160	±30°	Zigzag (20–42°)^f)^	12–16	31–77^e)^	3.8–16	9–19
Meniscus	70–80	<1	17	150‐600	0–90°	Waves/helical (19–27°)	15–41	10–300	1–18	20–50
Skin	60–80	2–4	∼20	60‐120	Bimodal (Langer lines)	Helical	∼10	50–150	10–30	30–70
Aorta	25–35	40–50	2–5	∼80	0–90°	Sine wave	50–100	3–5	0.3–1.0	50–100

^a)^ There is large variability between SFT types, species, and anatomical regions, and the mechanical properties tested under different protocols, mechanical definitions, orientations, and sample processing;

^b)^ Data were taken from.^[^ ^10^, ^16^, ^17^, ^23^, ^24^, ^25^, ^26^, ^27^, ^28^, ^29^, ^30^, ^31^, ^32^, ^33^, ^34^, ^35^, ^36^, ^37^, ^38^, ^39^, ^40^, ^41^, ^42^, ^43^, ^44^, ^45^, ^46^, ^47^, ^48^ ^]^ Tendons and ligaments data are averaged across multiple tissue types, detailed in;^[^ ^9^ ^]^

^c)^ % dry weight;

^d)^ Taken in the linear region;

^e)^ Single lamella properties ‐tested in the fiber direction;

^f)^ Changes in the radial position.

When comparing SFTs to classical composites, key factors such as fiber fraction, fiber orientation, and the stiffness disparity between fibers and the matrix—often spanning 2–3 orders of magnitude—create a vast design space. However, unlike synthetic composites, both the fibers and matrix in SFTs can undergo large deformations. Additionally, while in engineered composites, an idealized fiber‐matrix interface is assumed based on the rule of mixtures, biological SFTs have more complex interfaces, which leads to combined matrix and interface failures.^[^ ^9^, ^10^ ^]^ These interfaces result in the effective mechanical behavior that is much lower than the theoretical sum of their constituents.

Collagen fibers do not contribute significantly to the initial portion of the stress–strain curve due to their crimped morphology and need for reorientation. This results in fiber‐level strains that are lower than the macroscale strain,^[^ ^4^ ^]^ providing robustness and reduced wear over time and enhancing fatigue resistance, especially in tissues that are subjected to large deformations.^[^ ^9^ ^]^

### 2.2 Fiber Fraction, Fiber Orientation, and Orientation Distribution

SFTs exhibit varying collagen volume fractions, which directly influence tissue stiffness. As the volume fraction increases, stiffness generally increases, given that collagen is the primary load‐bearing component in SFTs. However, due to the hierarchical nature of collagen, directly measuring the fiber fraction is challenging. To provide a qualitative correlation with SFT stiffness, Table 1 presents the percentage of building blocks by dry weight along with representative fibril diameters.

Fiber orientation is a key determinant of deformation behavior. Tissues with fibers aligned along the loading direction tend to exhibit greater stiffness. Thus, tissues subjected to multiaxial loading exhibit more varied fiber orientations, enabling them to deform more easily as fibers gradually reorient toward the loading axis. For example, collagen fibers in tendons and ligaments align predominantly uniaxially with the loading direction, while tissues like skin and blood vessels, which undergo multiaxial loads, display more dispersed fiber orientations. This distribution allows greater deformation as fibers reorient toward the load, enabling mobility within the tissue and preventing damage within the physiological range. In the annulus fibrosus (AF), collagen fibers reorient by 17 degrees under a 10% physiological strain.^[^ ^49^ ^]^

Although the skin is relatively isotropic, it can develop pronounced anisotropy under tension as collagen fibers reorient toward the loading direction. As these fibers align, they contribute to the tissue's characteristic nonlinear J‐shaped mechanical behavior.^[^ ^50^ ^]^ In the skin, collagen fibers exhibit a bimodal orientation but with a wide distribution, leading to fiber buckling and gradual alignment in the loading direction under tension. Lynch et al.^[^ ^51^ ^]^ observed that in the heel region of the skin, the fraction of fibers aligned with the loading direction increases nonlinearly as the tissue stretches. Fibers oriented perpendicular to the direction of the applied force must first buckle and bend before they can reorient and carry the load.

Fiber realignment plays a crucial role in SFTs with highly dispersed fiber distributions, such as skin, fetal membranes, and pericardium, in governing tear behavior and blunting crack propagation, thereby enhancing fracture toughness.^[^ ^52^ ^]^ Bircher et al.^[^ ^52^ ^]^ showed that at the crack tip, collagen fibers reorient perpendicularly to the direction of the crack, resulting in local densification that mitigates crack propagation. This shielding effect significantly enhances defect tolerance, enabling some tissues to resist crack growth from defects several millimeters in size—achieving fracture toughness levels up to 100 times greater than synthetic polymers.

Similarly, pig skin exhibits remarkable tear resistance under shear due to local collagen realignment in the direction of principal tension, which mitigates crack propagation at the crack tip.^[^ ^53^ ^]^ This realignment mechanism has also been observed in chemically fixed bovine pericardium used in prosthetic heart valve leaflets. Notably, this adaptive realignment response has not yet been successfully reproduced in synthetic polymers.^[^ ^54^ ^]^

Collagen fiber reorientation is also linked to pathological conditions. In aneurysmatic abdominal aortas (AAA), collagen fibers show increased out‐of‐plane dispersion, which obscures the normal wall structure and compromises mechanical function. This loss of organization makes AAA tissue more susceptible to tear failure.^[^ ^55^ ^]^ A similar loss of structural anisotropy is observed in post‐menopausal vaginal tissue.^[^ ^56^ ^]^ These examples underscore the importance of collagen fiber orientation in maintaining mechanical integrity and resistance to failure.

### 2.3 Crimping

Another key structural motif is collagen fiber crimping, which drives the J‐shaped mechanical behavior of SFTs.^[^ ^34^, ^57^ ^]^ In the toe and heel regions, the microscopic crimp straightens without stretching the fibers, a process mediated by GAGs and PGs, providing wear protection.^[^ ^4^, ^58^ ^]^

Crimp morphology varies widely across tissues and functions, with wavelengths ranging from 10 to 200 µm.^[^ ^59^ ^]^ In tissues subjected to unidirectional loads, such as tendons, ligaments, the AF, and the meniscus, the crimp typically appears as a planar zigzag or parallel waveform. In contrast, tissues such as skin and blood vessels, which experience multiaxial loading, often display helical or sinusoidal crimps, reflecting the more dispersed and non‐parallel alignment of collagen fibers.^[^ ^9^ ^]^ Interestingly, regions of tendons and ligaments exposed to multiaxial loads also exhibit helical crimp patterns.^[^ ^60^ ^]^ The straightening length of the helicoid crimp is greater than that of the parallel wavy or zigzag crimp, enabling tissues with helical crimp morphologies to accommodate larger deformations.

Crimp is primarily found at the fascicle and fiber levels, acting as a flexible hinge for these wavy structures. However, Franchi et al.^[^ ^61^ ^]^ identified periodic sharp kinks at the nanometer scale—referred to as fibrillar kinks—within the fibrils of tendons and ligaments. These fibrils, aligned in parallel, undergo abrupt directional changes at the apex of each crimp, resembling origami folds. Unlike typical kinks, these function more like knots, creating regions of enhanced flexibility within the robust fibrils. This flexibility is achieved through adjustments in their alignment and microfibrillar organization, allowing localized relaxation of structural constraints and improving their ability to recoil.

Jan and Sigal^[^ ^62^ ^]^ demonstrated gradual collagen fiber recruitment in the eye under increased intraocular pressure (IOP) using polarized light microscopy (PLM). They identified two distinct recruitment regimes in the lamina cribrosa (LC) and peripapillary sclera (PPS), showing that different fractions of fibers are recruited at varying IOPs. This recruitment may act as a protective shielding mechanism, where the PPS relieves tension from the LC, reducing the fraction of fibers under load at elevated IOPs.

The importance of crimping is also evident in pathological conditions. For instance, in aneurysmal abdominal aortas (AAA), some collagen fibers lose their crimp, appearing straighter and thicker than in healthy aortas.^[^ ^55^ ^]^ These observations highlight the critical role of fiber crimping in preventing tears and maintaining the structural integrity of SFTs.

### 2.4 Hierarchy and Multiscale Materials

Nature employs hierarchical structures and multiscale material organizations to optimize mechanical performance. Collagen Type I, one of the most extensively studied hierarchical materials, exemplifies this strategy and has been comprehensively reviewed in prior studies.^[^ ^2^, ^9^, ^10^ ^]^ These hierarchical structures facilitate mechanical responses and structural adaptations across multiple scales, effectively mitigating strain on internal components. For example, in tendons, the strain experienced by individual fibrils is ≈40% lower than the overall strain applied to the tendon.^[^ ^4^ ^]^

This multiscale mechanical behavior has been elucidated using advanced complementary techniques such as multiphoton microscopy, polarized light microscopy (PLM), and small‐angle X‐ray scattering (SAXS).^[^ ^22^, ^50^, ^51^, ^53^, ^62^, ^63^, ^64^, ^65^, ^66^, ^67^ ^]^ These tools are used in situ to capture structural changes during mechanical loading across various hierarchical levels—such as crimp straightening, fiber reorientation, and molecular sliding—thereby offering mechanistic insight into the structure–function relationship.

For instance, in tendons, stretching initially leads to the gradual straightening of collagen fascicles.^[^ ^67^ ^]^ In this toe region, the matrix—composed primarily of PGs—dominates the mechanical response, allowing for significant deformation even under low loads. As loading increases, the tissue transitions into the heel region, where molecular kinks are straightened, followed by simultaneous stretching of the collagen triple helix and molecular sliding in the linear regime.

Recent studies have shown that matrix components at multiple scales—including those between sub‐tendons and fascicles—also contribute to the sliding mechanisms observed in the toe region, underscoring the matrix's role across hierarchical levels.^[^ ^68^, ^69^ ^]^ However, the precise mechanisms of force transmission between these levels remain unresolved.^[^ ^70^ ^]^ Data on more complex soft tissues is even more limited, highlighting the need for further investigation.

In addition to hierarchical organization, SFTs integrate multiscale fiber structures, often comprising both nanofibers and microfibers.^[^ ^71^, ^72^ ^]^ Although the rationale for combining multiple length scales is not fully understood, there is evidence suggesting that nanoscale fibers contribute to toughness, while microscale fibers enhance stiffness.^[^ ^71^, ^73^, ^74^ ^]^

Another important factor is the force transmission across scales, which is influenced by overall tissue deformations. When the substrate undergoes stretching, it leads to nonlinear macroscopic stiffening and provides dual advantages: at the macroscale, it accommodates the necessary mechanics for tissues, including nonlinearity, anisotropy, and time‐dependent behavior, enabling load‐bearing capacity and strength. Simultaneously, at the microscale, a mechanically favorable environment for resident cells supports appropriate mechanotransduction by providing suitable local stiffness and structural topology. This ability to modulate mechanics across scales is a critical feature of hierarchical SFTs and cannot be achieved with homogeneous materials. The microscopic stiffness at the cellular scale is significantly lower than macroscopic stiffness, which is itself lower than that of isolated fibers by several orders of magnitude. This creates a gap between the biomechanical requirements of cells and those of the tissue. Some tissues, such as articular cartilage, bridge this gap using the pericellular matrix (PCM), which mediates between the cells and the extracellular matrix (ECM).^[^ ^75^, ^76^ ^]^

It is worth emphasizing that strain transmission across different scales in SFTs is typically non‐uniform. This implies that deformations at smaller length scales can exhibit substantial variation and are not consistently proportional. Consistency is only achieved across a critical length scale when averaged. Consequently, the strain transfer is not solely dependent on scale. However, it is also influenced by the specific characteristics of the tissue since the specific topology and non‐collagenous matrix are also crucial in this process, and this data remains incompletely characterized.^[^ ^77^ ^]^

### 2.5 Water, Interface Mechanics, and Hydrophilicity in SFTs

All SFTs exist in an aqueous environment, where the presence of water plays a crucial role in both biological functions and mechanical behavior.

**Figure 2:**
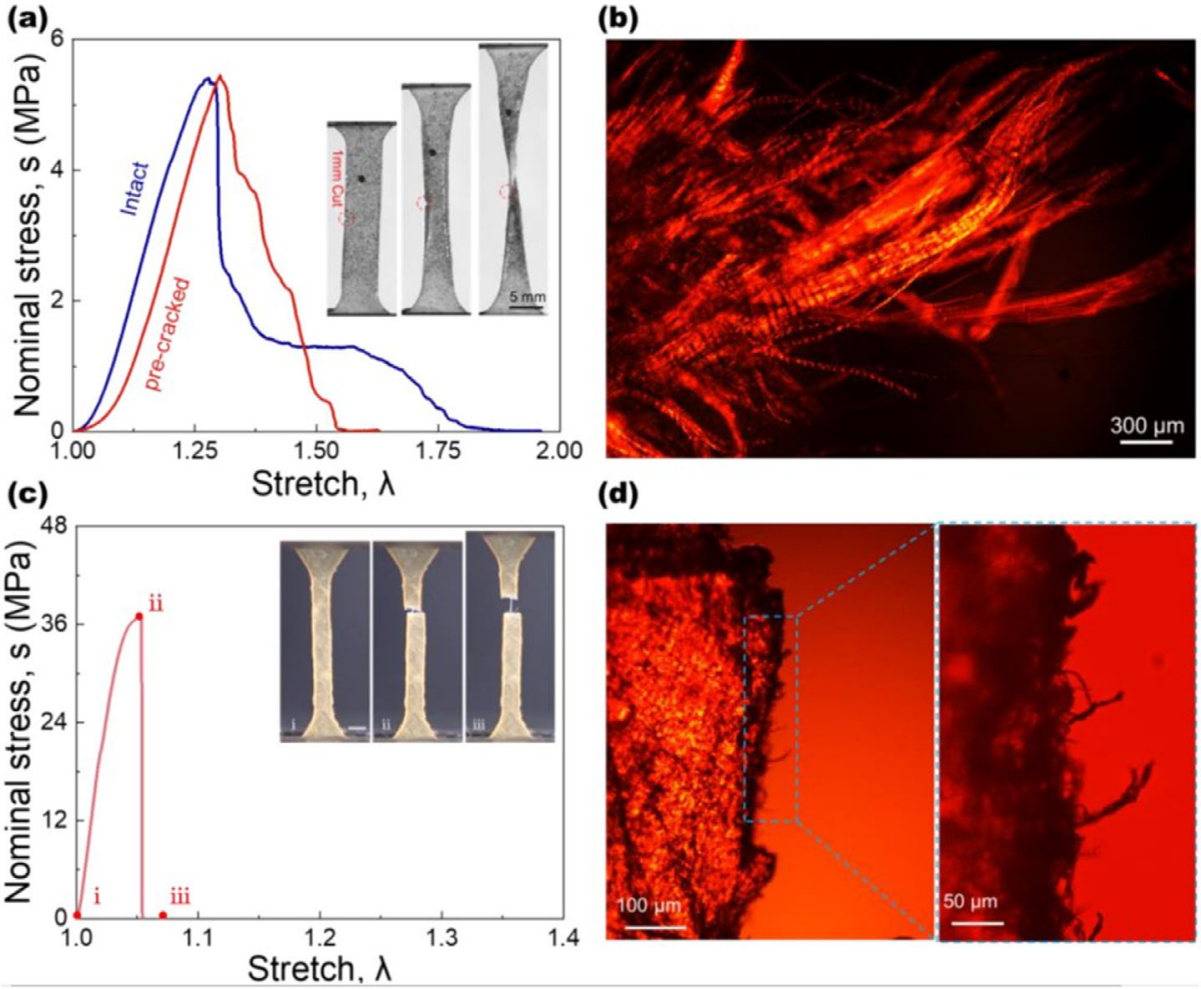
Effects of cracking and dehydration on the deformation behavior of bovine pericardium (BP). a) Stress–stretch curves of BP samples with and without a precut, with corresponding snapshots of single‐edge‐notched samples at various stages of deformation. b) Polarized light micrograph of the ruptured surface in a pre‐cracked sample, showing evidence of collagen fiber pull‐out. c) Stress–stretch curve of a dehydrated BP sample, accompanied by snapshots illustrating progressive deformation states. d) Polarized light micrograph of the rupture surface in the dehydrated sample. Reproduced under the terms of CC BY‐NC license.^[^ ^54^ ^]^ © 2023, Zeng et al., published by AAAS.

The mechanical properties of tissues can change significantly between dry and wet conditions (**Figure** **2**), as well as in response to variations in osmolar concentration.^[^ ^10^, ^54^, ^78^ ^]^ Water contributes to the viscoelasticity, poroelasticity, and shock‐absorbing capacity of the tissue. For instance, elastin achieves its extreme stretchability only when hydrated; thus, water confers flexibility to elastin and elastic fibers. Meanwhile, negatively charged PGs and GAGs absorb water and bind to collagen fibrils, mediating their sliding and stretching behavior.^[^ ^9^, ^18^, ^20^, ^21^, ^22^, ^78^, ^79^, ^80^, ^81^ ^]^ A fundamental hydration‐dependent mechanism is hydrogen bonding, which governs mechanical behavior by allowing weak interfacial connections and enabling reversible sliding between fibers across multiple hierarchical levels.^[^ ^9^, ^10^ ^]^ Therefore, the presence of water is essential for all components to maintain their mechanical properties and fulfill their biological roles.

**Figure 3:**
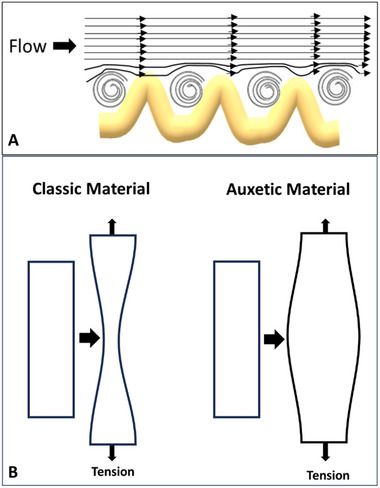
a) Schematic illustration of drag reduction on a crimped collagen‐textured surface. b) Comparison of auxetic and conventional materials under uniaxial tension, highlighting differences in transverse deformation.

Internal interfaces in SFTs are predominantly hydrophilic. Moreover, their complex architecture introduces considerable topological roughness—arising from collagen fiber crimping, hierarchical organization, and other intrinsic features. This multiscale surface roughness, coupled with immersion in aqueous environments, promotes **superhydrophilicity** (extreme wettability). Such conditions are likely to enhance fluid transport, reduce drag, and facilitate inter‐fiber sliding at different scales.^[^ ^82^ ^]^ The drag‐reducing effect of textured, wet surfaces is associated with micro‐vortices forming within surface grooves. These vortices enable fluid to “roll” across the interface, acting like tiny wheels, thus minimizing frictional resistance and boundary layer adhesion.^[^ ^83^ ^]^(**Figure** **3**A).

Finally, as discussed earlier, biological SFTs exhibit complex internal interfaces that rely on sacrificial bonding mechanisms—such as hydrogen bonds and van der Waals interactions. These mechanisms result in combined matrix–interface failure,^[^ ^9^, ^10^ ^]^ in contrast to the interfacial crack deflection typically seen in hard natural composites.^[^ ^84^ ^]^

### 2.6 Additional Structural Motifs

***Auxetics***. The term *auxetics* originates from the Greek word “αυχητικoς” (read: auxetikos), meaning “that which tends to increase.” It describes materials exhibiting a negative Poisson's ratio—expanding laterally when stretched rather than narrowing (Figure 3B). Auxetic behavior has been reported in several biological tissues, including skin,^[^ ^85^, ^86^ ^]^ blood vessels,^[^ ^85^, ^86^ ^]^ tendons,^[^ ^87^ ^]^ and intervertebral discs.^[^ ^88^, ^89^, ^90^ ^]^ However, Skacel et al.^[^ ^91^ ^]^ reevaluated earlier findings and concluded that arteries do not display true auxetic behavior.

That said, SFTs exhibit non‐standard Poisson's ratios under uniaxial tension due to their complex fibrous architectures. Unlike homogeneous materials, the transverse deformation in SFTs is driven by fiber buckling and reorientation under tensile strain.^[^ ^51^ ^]^ These tissues are characterized by large deformations and nonlinear, strain‐dependent Poisson's ratios.

One key mechanical benefit of auxetic‐like behavior is crack closure under tension rather than propagation. Although the term “auxetic” may not be strictly applicable to soft biological tissues, their transverse strain response offers important biomechanical insights. For instance, radial or transverse fibers that interconnect circumferential or axial fiber networks may resist narrowing and delamination, thereby helping to prevent axial or circumferential tearing.^[^ ^92^ ^]^

***Gradients***. Natural materials frequently exhibit functional gradients and heterogeneities that support the formation of robust, high‐performance biological structures, as comprehensively reviewed by Liu et al.^[^ ^93^ ^]^ Stiffness gradients are widely observed in biological interfaces, such as tendon‐to‐bone insertions^[^ ^94^ ^]^ and intervertebral discs.^[^ ^47^, ^88^ ^]^ These gradients help to reduce stress concentrations, alleviate mechanical singularities, and improve bonding across dissimilar tissue types, enhancing both durability and fatigue resistance.

## 3 Mechanical Behaviors

SFTs exhibit exceptional mechanical properties that enable them to function under physiologically demanding conditions, such as repetitive joint motion, pressure fluctuations in blood vessels, and load‐bearing in musculoskeletal systems over an extended period. These properties include large deformation with strain stiffening, exceptional toughness, and remarkable fatigue and damage tolerance. SFTs achieve an ideal balance of stiffness (resistance to elastic deformation), strength (resistance to plastic deformation), and toughness (resistance to fracture). These extraordinary capabilities arise from the unique structural motifs and designs discussed in the previous section.

Notably, a single structural motif often contributes to multiple mechanical behaviors. While large deformations are inherently linked to the mechanisms responsible for extreme toughness in SFTS, we separated these aspects in this section to clarify their underlying principles and facilitate the development of biomimetic applications.

### 3.1 Strain Stiffening and Large Deformations

**Figure 4:**
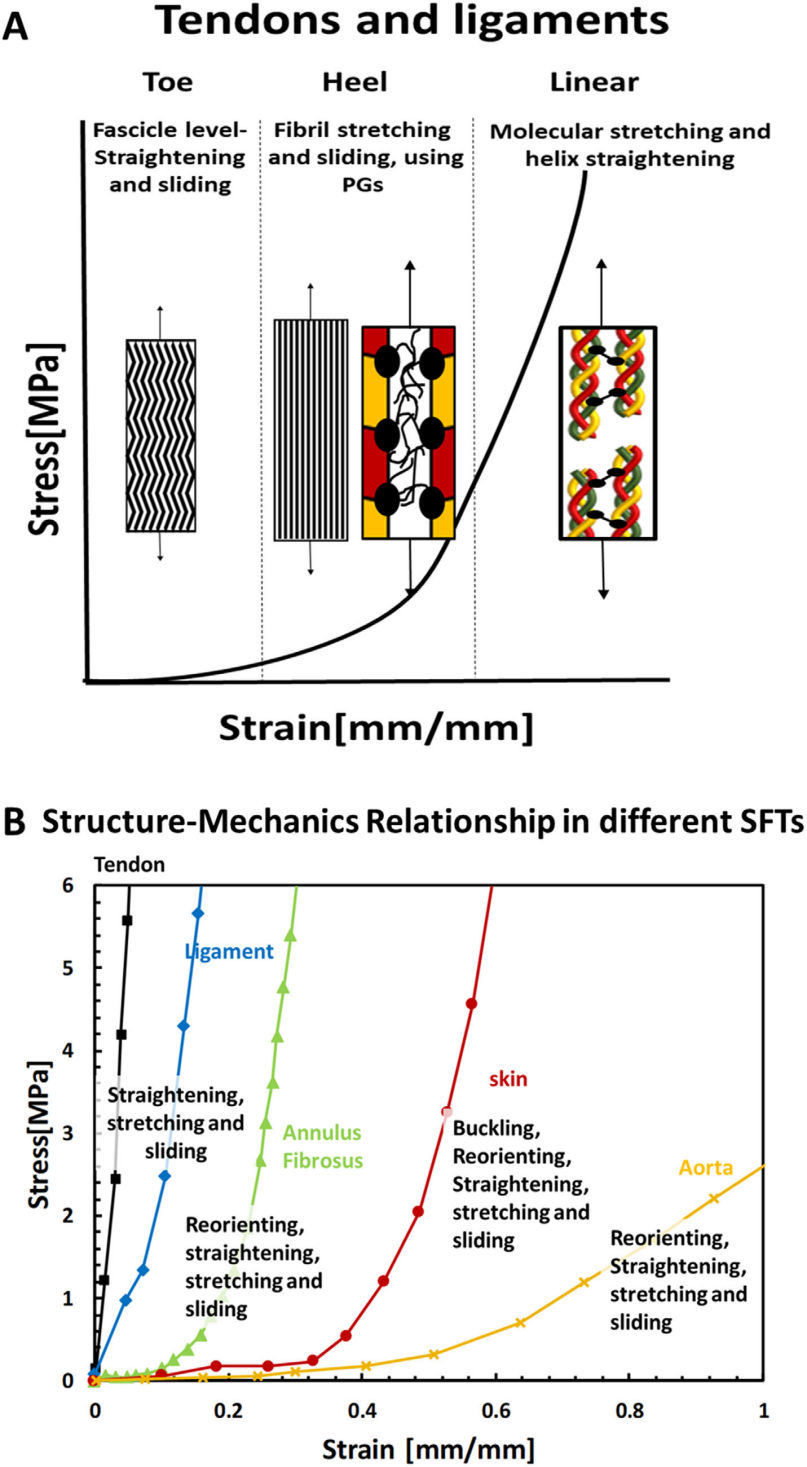
a) Schematic illustration of the J‐shaped stress–strain response and underlying deformation mechanisms in tendons and ligaments. In the toe region, microscopic crimping within fascicles is progressively straightened. As fibrils uncrimp, they begin to bear load, initiating simultaneous sliding and stretching processes mediated by PGs, characteristic of the heel region. In the linear region, load is supported by molecular‐level mechanisms, including helix straightening and stretching of covalent cross‐links.^[^ ^4^ ^]^ b) Representative uniaxial tensile behaviors of various soft fibrous tissues and associated deformation mechanisms, including tendon and ligament, annulus fibrosus, skin and aorta^[^ ^29^, ^95^, ^96^, ^97^ ^]^ These curves illustrate typical mechanical behaviors; however, variations in experimental protocols, orientations, anatomical origin, and tissue type can lead to substantial differences in observed behaviors. Reproduced under the terms of the CC‐BY license^[^ ^9^ ^]]^ 2022, Sharabi, published by Frontiers.

SFTs are characterized by J‐shaped behavior with strain stiffening and can be divided into three distinct regions: toe, heel, and linear regions (**Figure** **4**A). This behavior is attributed to the composite and hierarchical organization of collagen fibers, which are the main loading elements of the tissues. The stiffening regime is related to the recruitment of collagen fibers to carry applied loads. This recruitment usually involves crimp straightening, fiber reorientation, or both (Figure 4B). We have compared the stress–strain behavior of four representative SFTs:^[^ ^9^ ^]^ tendons and ligaments,^[^ ^95^ ^]^ annulus fibrosus (AF) (stretched in the fiber direction),^[^ ^96^ ^]^ skin,^[^ ^97^ ^]^ and the aorta.^[^ ^29^ ^]^ These curves illustrate typical J‐shaped behaviors under uniaxial stretching. It is important to note that variations in experimental protocols, anatomical origin, and tissue type can lead to considerable differences in the observed mechanical behaviors and the data presented here are representative. To further illustrate the structure–mechanics relationship, we have added descriptions of the structural adaptations observed at multiple scales for each tissue type based on the available literature (tendons and ligaments,^[^ ^4^ ^]^ AF,^[^ ^49^ ^]^ skin^[^ ^50^, ^51^, ^65^ ^]^ and the arterial wall^[^ ^98^ ^]^).

Tendons and ligaments exhibit a distinct J‐shaped behavior with a steep slope compared to other fibrous tissues. In this toe region, when the stretching commences, the crimping in the fascicles is straightened, and the mechanical properties are primarily governed by the matrix components, such as elastin and PGs, resulting in relatively significant deformation even under low loads. In the heel region, the stiffness increases considerably with extension.^[^ ^4^ ^]^ In this region, the kinks within the gaps between fibrils straighten out.^[^ ^67^ ^]^ Simultaneously, fibril sliding and elongation occur, regulated by the PG and GAGs matrix in a finely tuned balance^[^ ^22^ ^]^ (Figure 4A).

In the linear region, the collagen triple helices and crosslinks stretch, eventually leading to a helix pitch change.^[^ ^4^, ^67^ ^]^ Furthermore, at the molecular and sub‐fibril level, an additional slipping occurs due to a deficiency in covalent cross‐linking between molecules in the fibrils.

**Figure 5:**
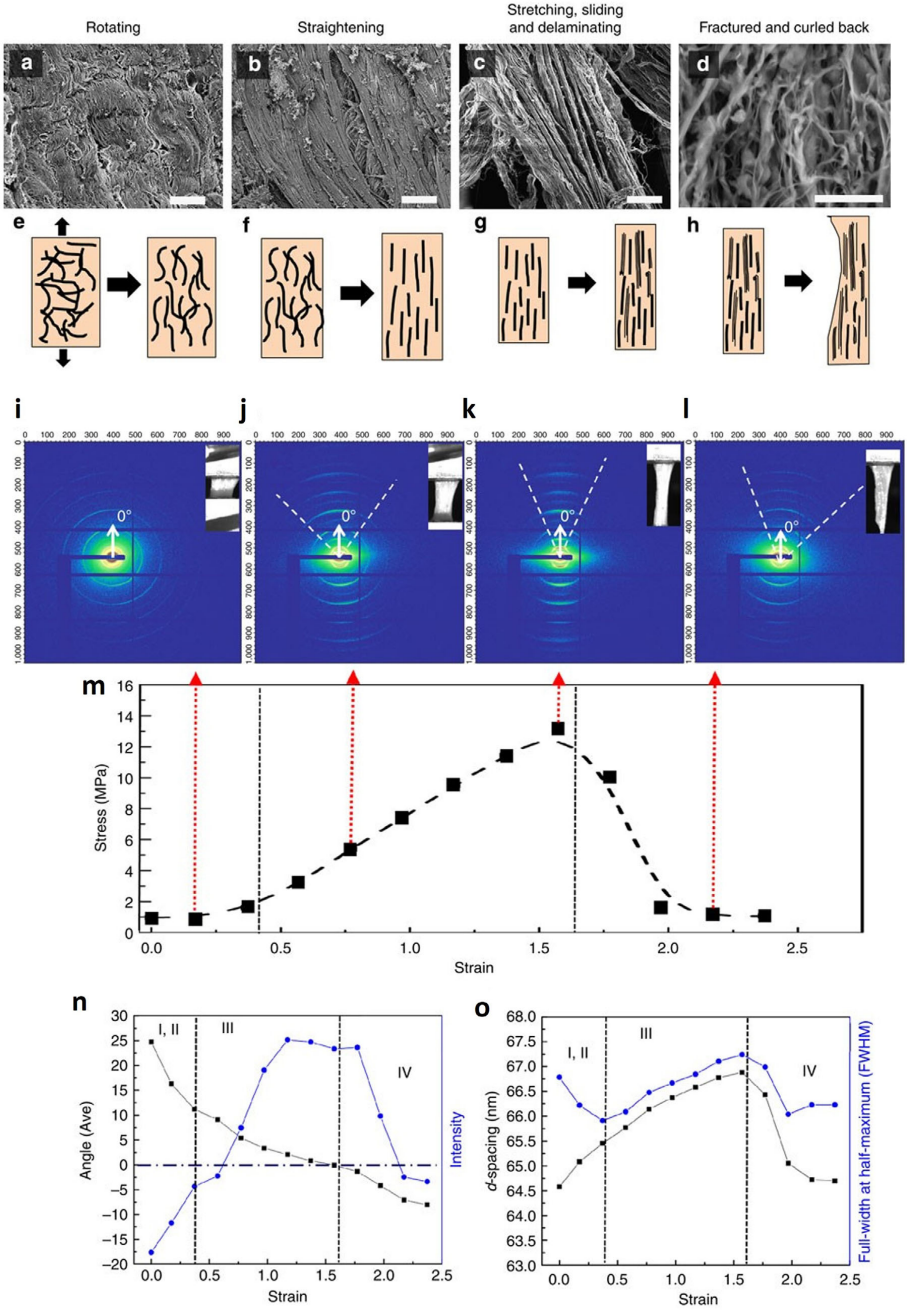
SEM and SAXS Analyses of Tensile Loading in Rabbit Skin. SEM images. a–d) and schematic drawings e–h) illustrate the four stages of tensile loading in rabbit skin: (a,e) collagen fibrils are curved and align with the tensile axis; (b,f) fibrils straighten and reorient toward the tensile axis; (c,g) fibrils stretch, slide, delaminate, and fully align; (d,h) fibrils fracture and curl back. SAXS analysis. i–l) Diffraction patterns show fibril orientations, with images of the sample at top‐right corners. (i) Collagen fibrils are randomly oriented to the tensile axis, as indicated by constant diffraction pattern intensity; (j) fibrils gradually align with the tension direction; (k) fibrils fully align with the tensile axis; (l) fibrils fracture and relax. m) Stress–strain behavior with the corresponding data points of I‐L. The stress–strain curve reveals four stages indicated in images A‐H: I‐II‐ toe and heel. III – linear and IV ‐fracture. n) Angle of normal to the tensile axis versus intensity of fibrils (as a function of strain, and o) *d*‐spacing and full‐width half‐maximum (FWHM) of fibrils as a function of strain. Reproduced under the terms of the CC‐BY license^[^ ^50^ ^]^ 2015, Yang et al., published by Springer Nature.

While the mechanisms of force transmission across hierarchical levels in tendons and ligaments remain unclear,^[^ ^70^ ^]^ the matrix is believed to play a central role. Additional collagen recruitment mechanisms were observed in tissues such as skin and blood vessels^[^ ^9^, ^10^, ^50^, ^98^, ^99^ ^]^ (Figures 4B and **5**). However, the intricate interconnections among different hierarchical levels under mechanical loading remain even less understood in these tissues, underscoring the need for further investigation. In skin and blood vessels, greater deformations are observed due to the simultaneous activation of multiple mechanisms—fiber realignment, straightening, buckling, stretching, and sliding—which enhances their functional adaptability (Figure 4B). The magnitude of deformation increases as more mechanisms are engaged. Beyond the mechanisms known for tendons and ligaments,^[^ ^4^ ^]^ additional and potentially distinct processes likely contribute to the ability of these tissues to undergo large deformations and exhibit complex stiffening responses.^[^ ^9^ ^]^

### 3.2 Toughness and Tear Resistance

One of the key strengths of SFTs is their remarkable ability to resist cracks and tears, endure millions of cycles of fatigue without failure, and achieve exceptional tearing toughness. These tissues experience only minor reductions in strength, even in the presence of **millimetric‐size** defects. In contrast, engineered materials for biomedical applications often struggle to withstand fracture and fatigue, limiting their performance. This section explores fracture behaviors in soft tissues to guide the development of superior biomimetic replacements and next‐generation engineered materials.

**Figure 6:**
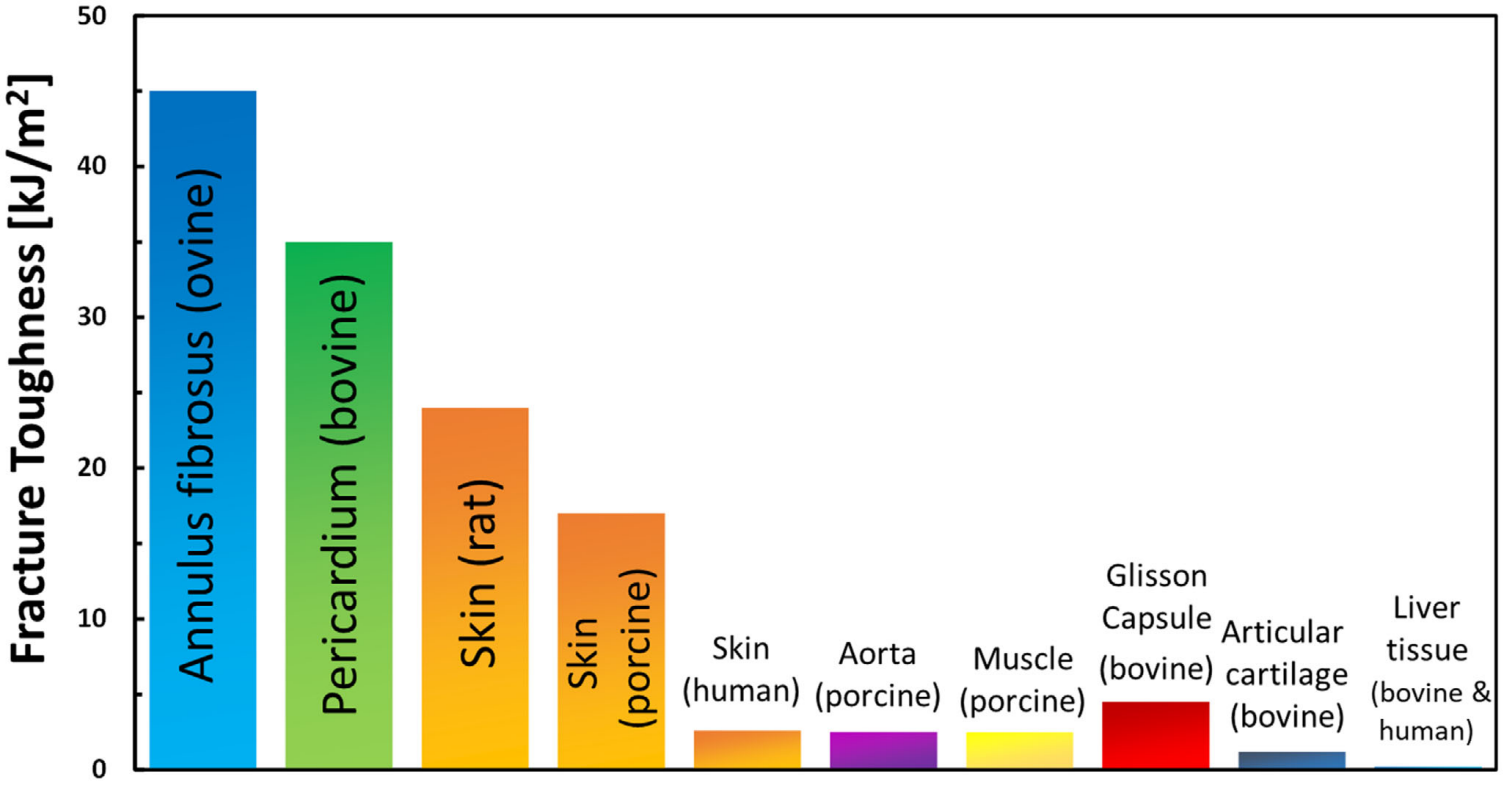
Representative values of fracture Toughness of Different Soft Tissues, based on.^[^ ^52^, ^54^, ^102^, ^103^, ^104^, ^105^, ^106^, ^107^, ^108^ ^]^ Notably, experimental methods are different between the different tissues, and the data represent average values.

Fracture toughness data for different SFTs remain limited in the literature. Although several examples are reported^[^ ^52^, ^54^, ^100^, ^101^, ^102^, ^103^, ^104^, ^105^, ^106^, ^107^, ^108^, ^109^ ^]^ (**Figure** **6**), tear behavior exhibits considerable variability, stemming not only from inherent biological heterogeneity but also from differences in testing methods, protocols, specimen orientations, and data analysis approaches. These discrepancies pose significant challenges for direct comparison across studies. Consequently, this review emphasizes general toughening mechanisms rather than specific quantitative values. A comparative analysis of fracture toughness methodologies in SFTs and their standardization is essential for further development of damage‐tolerant materials.

In contrast to hard biological materials such as nacre and bone,^[^ ^10^, ^84^, ^110^ ^]^ limited information is available regarding the structural mechanisms that impart damage tolerance and fracture resistance to SFTs. Recently, flaw‐insensitive mechanisms have been identified in tissues such as amnion,^[^ ^79^ ^]^ Glisson's capsule,^[^ ^52^ ^]^ knee meniscus,^[^ ^101^ ^]^ vagina,^[^ ^111^, ^112^ ^]^ and bovine pericardium^[^ ^54^ ^]^ using combined multiscale imaging and mechanical testing. These works begin to shed light on the structure‐function relationship and structural motifs that contribute to the incredible fracture resistance of SFTs.

**Figure 7:**
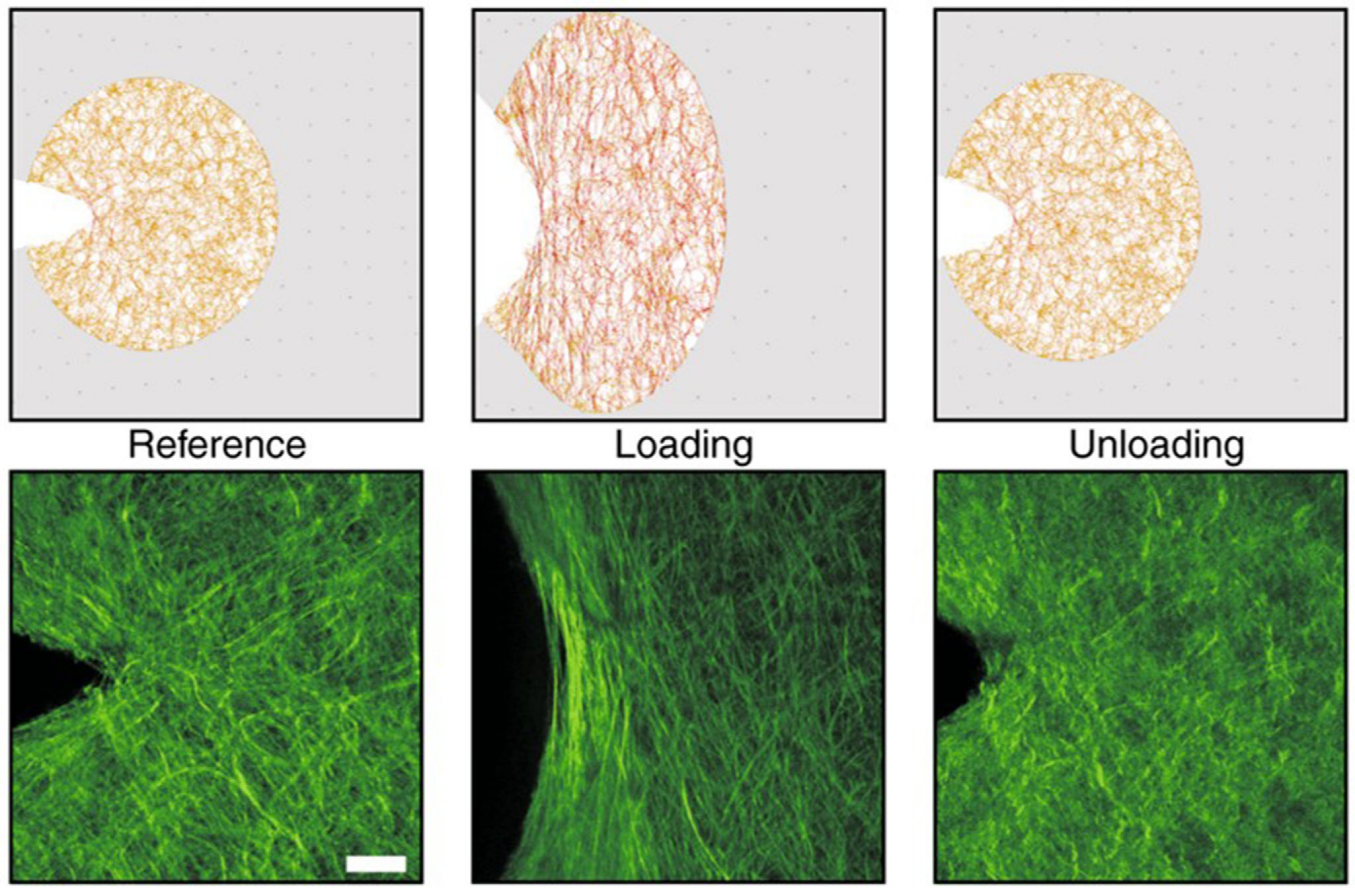
Near‐field analysis of notch‐like defects in SFTs. The morphology of the collagen fiber network and its localized alignment under mechanical loading. Upon unloading from subcritical loading, the near‐field fiber distribution is reversible and returns to its original configuration. The increased Second Harmonic Generation (SHG) intensity at the notch tip indicates a highly localized response, with significant collagen network densification (compaction) near the tip (scale bar: 50 µm). Reproduced under the terms of the CC‐BY license,^[^ ^52^ ^]^ 2019, Bircher et al., published by Springer Nature.

Bircher et al.^[^ ^52^ ^]^ showed that collagenous tissues undergo minimal strength loss despite millimetric defects. This is more evident in tissues with highly dispersed collagen fibers (e.g., skin, blood vessels), which impede crack propagation. However, processes near the crack tip are highly localized and lead to brittle behavior with limited energy dissipation. These tissues, while brittle and lacking long‐range stress redistribution, remain immune to large defects (**Figure** **7**).

Unlike highly dispersed fibrous tissues, tendons, with their uniaxially aligned fibers, rely on multiscale organization for damage tolerance. At the microscale, fibril strain is fully recoverable, whereas interfibrillar sliding only partially recovers. This incomplete recovery suggests that damage originates in the interfibrillar matrix, with irreversible sliding as a key mechanism. As a result, tendons exhibit an elongated toe region at the fascicle level and a reduced linear modulus at the macroscopic scale.^[^ ^113^ ^]^ Two tendon types exist: positional tendons (e.g., extensors), which operate under low loads, and energy‐storing tendons (e.g., flexors), which store and release strain energy. Their mechanical behaviors differ markedly: extensor tendons tolerate high plastic deformation, while flexors are optimized for elasticity.^[^ ^114^, ^115^ ^]^

Much less is known about soft tissue fatigue under cyclic stretching—an essential consideration for biomedical design.^[^ ^54^ ^]^ Fatigue in SFTs was investigated in tendons and ligaments,^[^ ^115^, ^116^, ^117^, ^118^, ^119^ ^]^ articular cartilage,^[^ ^120^ ^]^ and heart valves.^[^ ^54^, ^121^, ^122^, ^123^, ^124^, ^125^, ^126^ ^]^ It was found to induce structural changes, finally leading to gross disruption of the collagen fiber arrays; however, the exact mechanisms have not yet been revealed.

In extensor tendons, cyclic loading produces kink‐band damage in collagen fibrils, underscoring a trade‐off between strength and fatigue resistance.^[^ ^115^ ^]^ Thornton et al.^[^ ^116^ ^]^ found similar behavior in ligaments, identifying distinct strain and damage mechanisms under static (creep) and cyclic (fatigue) loading. Both types of loading resulted in a reduced linear modulus and an elongated toe region, with fiber and matrix damage. However, fatigue caused more severe damage than creep, as it led to a greater reduction in modulus and faster damage accumulation.

As with static fracture, multiscale architecture underpins fatigue resistance in tendons and ligaments. In highly deformable tissues like heart valves, fatigue is governed primarily by fiber realignment and straightening.^[^ ^54^ ^]^

Significantly, chemically fixed biological tissues exhibit structure–mechanics relationships that are similar to those of native SFTs,^[^ ^54^ ^]^ proving that these relationships are intrinsic material properties independent of the biological context. This insight is particularly valuable from a materials science perspective, as it allows emulation of these relationships in synthetic material systems.

**Figure 8:**
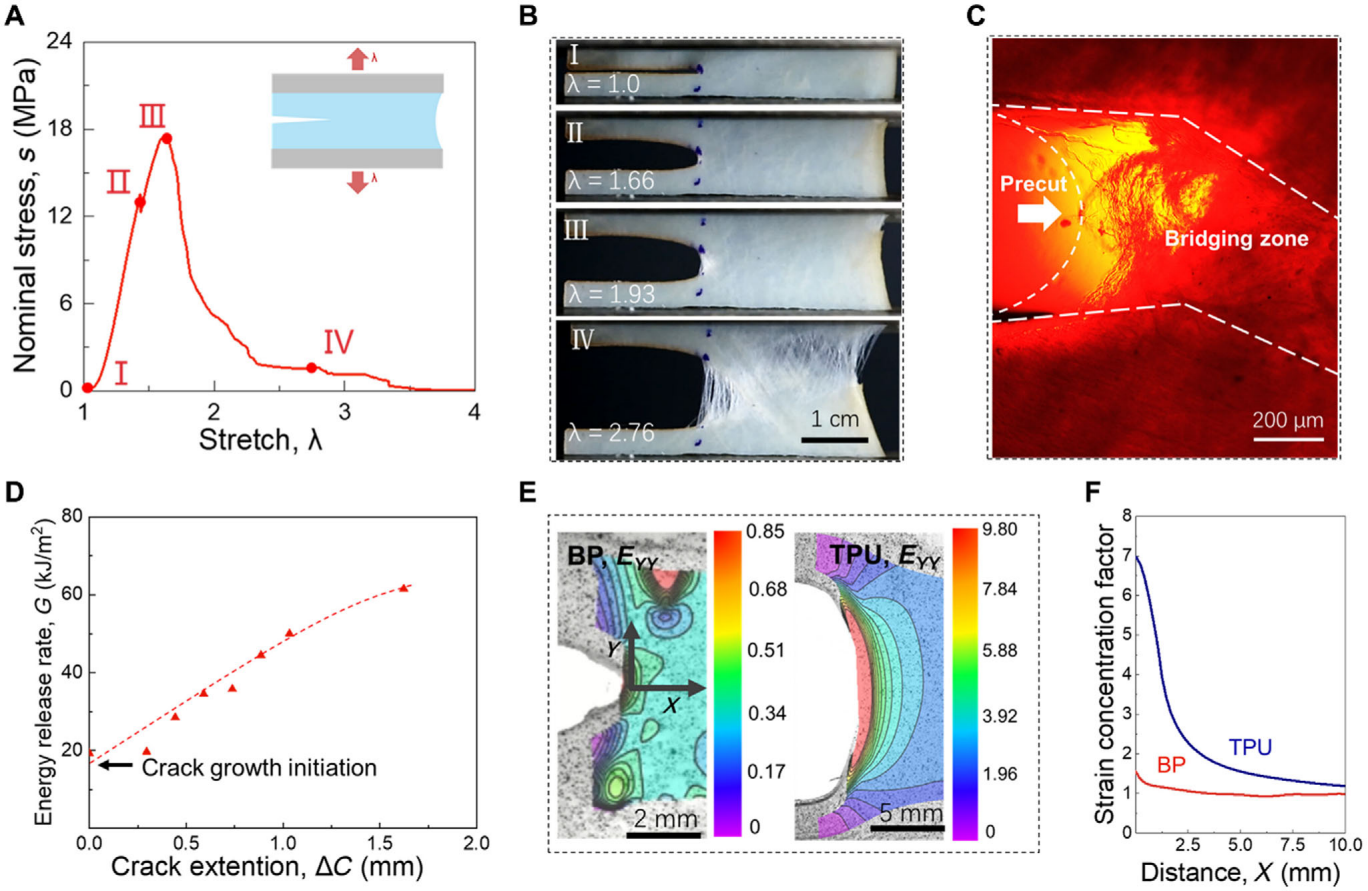
Fracture of chemically fixed bovine pericardium (BP). a) stress–stretch curve: in the initial state (I), the sample is undeformed. As stretching begins, the crack starts to open, and a small white zone appears at the crack tip (II), indicating initial crack growth. At this stage, stress doesn't drop immediately because collagen fibers in the white zone can still bear the load. As stress increases to its peak (III), the crack propagates, enlarging the white zone and further increasing stress. When the stretch reaches 2.76, the collagen fibers rupture and pull out in several regions, causing the stress to drop nearly to zero (IV). This stretch value is comparable to the rupture stretch of samples without precuts. b) Sequential snapshots of a membrane with a precut at different deformation stages. c) Polarized light micrograph showing the crack tip. d) Resistance curve illustrating crack propagation. e) Strain contour E_YY_ observed via Digital Image Correlation (DIC), comparing BP and Thermoplastic Polyurethane (TPU). f) Strain E_YY_ concentration factor as a function of distance X from the crack tip in the undeformed state. Reproduced under the terms of CC BY‐NC license.^[^ ^54^ ^]^ © 2023, Zeng et al., published by AAAS.

Zeng et al.^[^ ^54^ ^]^ have investigated chemically fixed bovine pericardium (BP) (commonly used for prosthetic heart valve leaflets) under static uniaxial tension and fatigue loading.^[^ ^54^ ^]^ These leaflets typically fail due to calcification rather than crack propagation. In contrast to polymeric thermoplastic polyurethane (TPU), BP leaflets resisted strain concentration at crack tips, as shown via digital image correlation (DIC). The TPU demonstrated resilience to flaws of ≈800 µm during static loading and 100 µm under fatigue loading. At the same time, BP samples displayed resistance to significant defects of 1 cm under both loading modes. This phenomenal fatigue‐tolerant behavior arose from the straightening, realignment, and sliding of collagen fibers rather than the stretching of collagen fibrils (**Figure** **8**).

## 4 Fracture Toughening Mechanisms in SFTs

Fracture toughness in biological materials is remarkable.^[^ ^1^, ^84^, ^110^ ^]^ While Launey et al.^[^ ^84^ ^]^ have comprehensively reviewed the toughness mechanisms in bone, the mechanisms responsible for toughness in SFTs remain largely unexplored. This section aims to investigate the structural motifs that contribute to the exceptional toughness and crack resistance observed in SFTs.

**Figure 9:**
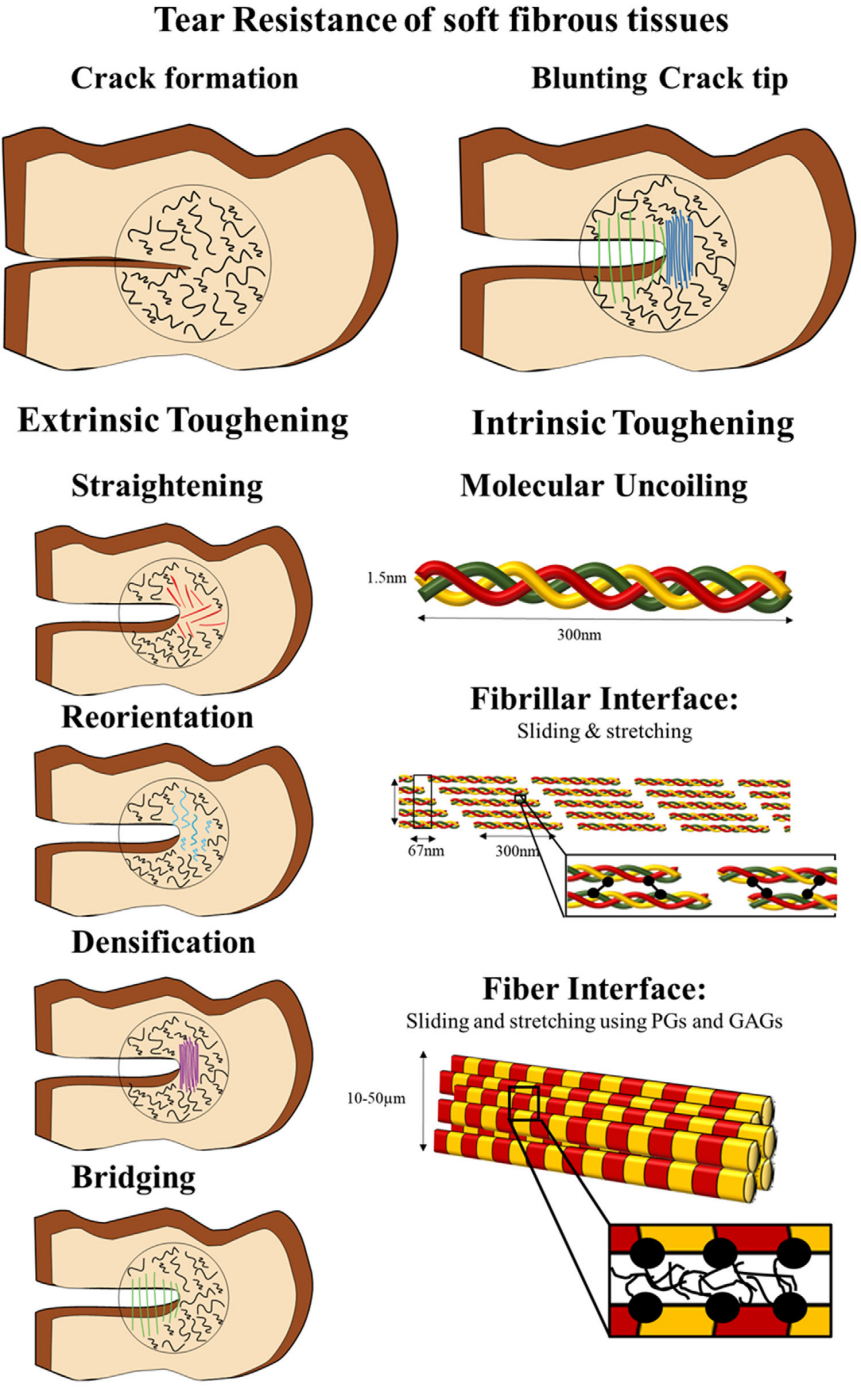
Tear resistance of SFTs. Illustration of extrinsic and intrinsic toughening mechanisms in SFTs.

Similar to bone, the toughness of SFTs is closely linked to their multiscale and hierarchical structure. Fracture arises from a dynamic interplay between a) **intrinsic toughening mechanisms**, occurring ahead of the crack tip, which primarily facilitate large deformations, and b) **extrinsic toughening mechanisms,** located mostly behind the crack tip, that blunt its progression. Intrinsic toughening mechanisms enhance the tissue's resistance to both crack initiation and propagation, much like the plastic deformation ahead of a crack tip in metals. In contrast, extrinsic toughening mechanisms involve microstructural processes that operate behind the crack tip, effectively reducing the forces driving crack growth^[^ ^84^ ^]^ (**Figure** **9**). To elucidate the multiscale mechanisms underlying this remarkable toughness, we divide our analysis into intrinsic and extrinsic toughening mechanisms.

### 4.1 Intrinsic Toughening (Shielding Mechanisms)

#### 4.1.1 Material Selection – Soft Tissue Building Blocks

Large deformations, driven by the structural components of tissues, are essential for enhancing fracture toughness. The properties of these constituents and their interactions play a significant role in determining the extent of deformation. For instance, collagen fibers accommodate larger deformations than molecules and fibrils,^[^ ^10^ ^]^ elastin can stretch up to 70%,^[^ ^15^ ^]^ and elastic fibers can extend to 100–220%.^[^ ^16^ ^]^ Consequently, elastin‐rich tissues, such as blood vessels and skin, display much greater deformation compared to tendons and ligaments.

#### 4.1.2 Hydrogen Bonds Breaking and Molecular Uncoiling

In the linear region of the stress–strain curve, deformations are primarily controlled by the hierarchical structure of collagen. This includes stretching and sliding at multiple scales, from fibril‐level sliding and stretching to the breaking of hydrogen bonds,^[^ ^9^ ^]^ as well as the uncoiling and stretching of collagen molecules,^[^ ^4^ ^]^ enabling large deformations in SFTs. As the tissue transitions to the plastic region, the deformations become irreversible.

#### 4.1.3 Crimp Straightening and Reorientation

In the toe and heel regions of the stress–strain curve, the straightening of collagen fiber crimp and subsequent fiber reorientation play a critical role in accommodating large deformations. These processes are largely reversible and are facilitated by elastin, which allows the tissue to return to its original configuration upon unloading. The extent and morphology of crimp vary across tissues and correlate with their mechanical roles. For example, skin and blood vessels exhibit greater deformation capacities than tendons and ligaments due to their more isotropic collagen architecture, dispersed fiber orientation, and enhanced capacity for realignment under load. Such structural features permit extended toe and heel regions in their mechanical responses, improving energy absorption and delaying the onset of irreversible damage.

#### 4.1.4 Chemical Potential, Hydration, and Inverse Poroelasticity

The aqueous environment provides a critical toughening mechanism in SFTs. Beyond facilitating fibril sliding and mediating the balance between stretching and sliding at multiple scales,^[^ ^9^ ^]^ hydration profoundly influences crack growth dynamics. Zeng et al.^[^ ^54^ ^]^ demonstrated that dehydration of bovine pericardium increased matrix stiffness, thereby limiting the transmission of tensile forces along collagen fibers over short distances. As a result, failure occurred through crack propagation, resembling classic material behavior, rather than by collagen fiber pull‐out in the neck region. Similarly, Bircher et al.^[^ ^52^ ^]^ showed that tissue strength depends on the chemical potential of the liquid environment. In particular, the tear resistance and critical elongation of amnion tissue were significantly impacted by dehydration, which can occur during prenatal surgeries. Complete restoration of tear resistance was observed upon rehydration.^[^ ^127^ ^]^

Tear resistance also depends on osmolarity. Studies on human amniotic membrane (hAM) demonstrate that changes in bath osmolarity alter collagen architecture or cross‐linking, thereby modulating tearing behavior.^[^ ^127^ ^]^ Ehret et al.^[^ ^79^ ^]^ found that inverse poroelasticity—fluid movement driven by osmotic gradients—enables tissues such as hAM, Glisson's capsule, and porcine pericardium to undergo large deformation and resist tearing. This reversible mechanism leads to localized densification at crack tips without permanent damage, as observed through multiphoton microscopy visualizing water movement near defects. In essence, collagen fibers provide tensile support, while interstitial fluid bears compressive loads and prevents collapse, enhancing toughness through dynamic, fluid‐structure interactions.

### 4.2 Extrinsic Toughening (Shielding Mechanisms)

Unlike bone,^[^ ^84^ ^]^ the extrinsic toughening mechanisms in SFTs operate through different mechanisms.

#### 4.2.1 Crimp Straightening, Fiber Reorientation, and Densification

Several intrinsic deformation mechanisms also operate as extrinsic shielding mechanisms. Specifically, the remarkable fracture toughness of SFTs is partly attributed to the nonlinear mechanical response of their collagenous fiber networks. Under mechanical loading, these fibers undergo strain‐induced alignment and lateral contraction near the crack tip, leading to localized material densification that effectively blunts and shields the advancing crack front.^[^ ^52^ ^]^

A fundamental mechanism that allows this behavior is the **blunting** of the crack tip using **Reorientation and Densification** motifs of collagen fibers. Pissarenko et al.^[^ ^53^ ^]^ demonstrated that strain concentration near the crack front in pig skin is alleviated through the local realignment of collagen fibers along principal stress directions. Simultaneously, the region adjacent to the crack reduces the high strain concentration by gradually recruiting collagen fibers. The sharp transition in strain near the crack tip also significantly increases the strains in the remaining segment along the crack path. This, in turn, diminishes the disparities between the maximum and minimum strain levels in the region, also reducing the strain concentration. Anisotropy is critical; in transverse samples, reduced strain gradients over a broader region imply that adjacent fibers contribute to shielding the crack. This finding indicates that collagen fibers adjacent to the crack contribute to mitigating strain concentration at the crack tip. Following the failure, the collagen fibers display clear irreversible damage, characterized by fibril delamination, loss of cohesion, and the formation of irregular, rugged structures.^[^ ^50^, ^53^ ^]^

The vaginal wall provides another example of these shielding processes during large deformations, such as childbirth.^[^ ^112^ ^]^ While over 80% of women experience vaginal tearing, the mechanisms remain poorly understood. McGuire et al.^[^ ^111^ ^]^ discovered that tears oriented in the hoop direction were significantly more blunted than those aligned axially, highlighting the critical role of anisotropy in this process. Like other soft tissues, vaginal tissue exhibits insensitivity to tears under sub‐failure loads due to collagen fiber straightening, sliding, and reorientation motifs, which help blunt tear propagation at the crack tip. Despite the more complex geometry and loading conditions observed during inflation tests, a similar trend was observed.^[^ ^111^ ^]^ In the ventral posterior region, collagen fibers exhibited significant straightening and alignment along the axial direction. In contrast, the mid and anterior ventral regions displayed some crimping and disorganization, yet a clear overall reorientation of the fibers toward the axial direction was evident. In the dorsal region, collagen fibers realigned tangentially to the axially oriented tear surface. Similar to other soft tissues, these collagen fibers formed an intrinsic shielding mechanism that mitigated crack propagation. Local variations in collagen fiber microstructure resulted in strain inhomogeneity, with initially dispersed and crimped fibers reorienting and straightening to enhance axial stiffness and hoop compliance. The tendency of fibers to align in the axial direction under biaxial stress, despite experiencing significantly greater hoop strains, may reflect a specific alignment pattern linked to vaginal physiology. However, the kinetics of fiber straightening, recruitment, and reorientation remain poorly understood.

#### 4.2.2 Bridging and Pull‐Out

**Figure 10:**
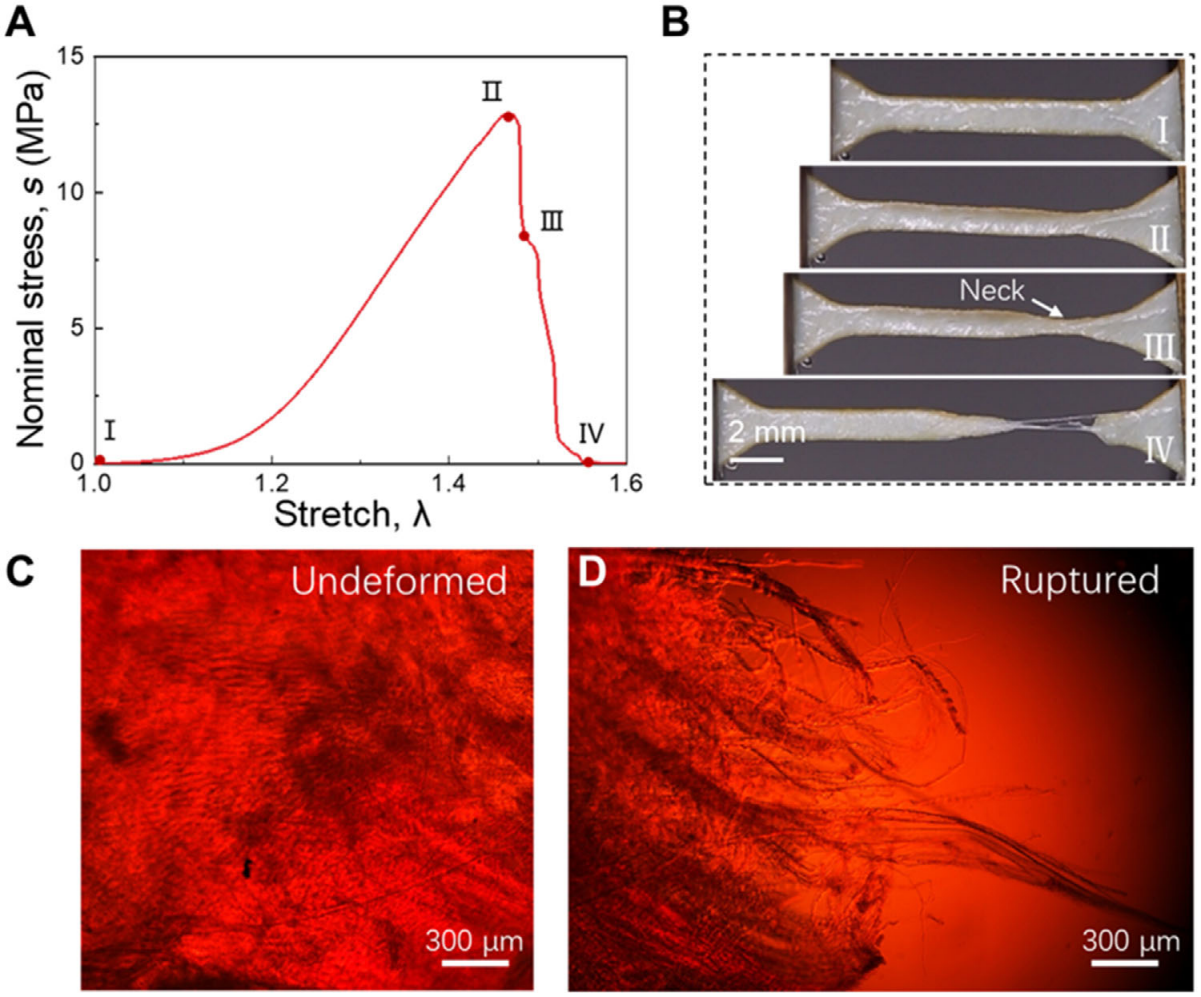
Tension and pullout of collagen fibers in chemically fixed bovine pericardium (BP). a,b) Stress–stretch curve and corresponding images depicting four sequential deformation states. c) Polarized light micrograph of the undeformed sample. d) Polarized light micrograph of the ruptured surface following failure. Reproduced under the terms of CC BY‐NC license.^[^ ^54^ ^]^ © 2023, Zeng et al., published by AAAS.

Unlike synthetic materials, notched soft tissues such as bovine pericardium exhibit failure modes similar to those of intact samples, characterized by the formation of a neck region with an extended pull‐out length of collagen fibers^[^ ^54^ ^]^ (**Figure** **10**). A similar behavior has been reported in the annulus fibrosus.^[^ ^101^ ^]^ The long pull‐out length indicates the dispersion of high tension and suggests that collagen fiber failure is primarily governed by statistically distributed factors, such as fiber strength or length, rather than localized high stress near the crack tip. This dispersion causes collagen fibers to rupture and pull out in multiple regions, creating a bridging zone that appears white to the naked eye. Consequently, cracks are deflected and often propagate in directions other than perpendicular to the applied load.^[^ ^54^ ^]^

## 5 Biomimetic Material Strategies: Recent Advances and Structural Inspiration

The previous sections provide a detailed overview of the mechanical behaviors of SFTs and the structural motifs that contribute to their function. However, they only partially elucidate the underlying mechanisms of SFTs due to gaps in the available information.

**Figure 11:**
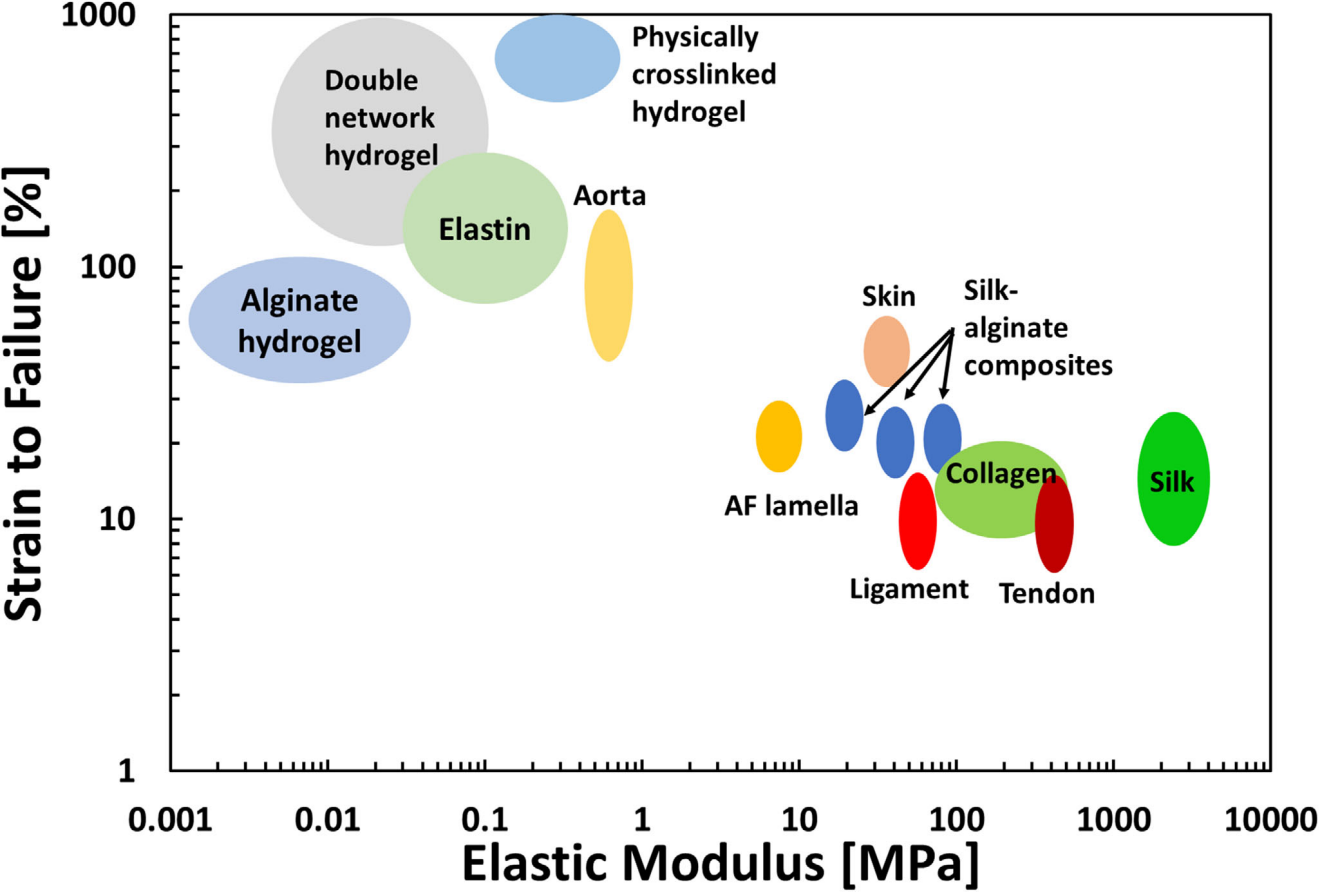
Ashbey diagram of several SFTs and representative synthetic biomaterials. Data is based on.^[^ ^9^, ^128^, ^156^ ^]^

This section reviews recent advancements in materials designed to replicate the complex behavior of SFTs, including those based on biomimetic principles and novel technological approaches. **Figure** **11** presents an Ashby plot comparing strain‐to‐failure versus elastic modulus among representative engineered biomaterials and various SFTs.^[^ ^9^, ^128^ ^]^ Notably, most of the currently developed materials focus on a specific mechanical property and, as a result, only partially address the comprehensive solution needed to replicate the full range of behaviors exhibited by a specific soft tissue. These materials are categorized based on structural features.

### 5.1 Soft Fiber‐Reinforced Polymer (FRP) Networks

Given the composite nature of SFTs, valuable insights can be gained from soft fiber‐reinforced polymer (FRP) networks. In FRPs, toughening arises from the pull‐out energy of the fibers, which is dissipated through the fracture of the exceptionally tough matrix surrounding the fiber bundles. When a transverse fiber bundle undergoes pull‐out, the matrix within the crossover region experiences significant deformation, leading to observable fibrillation. Due to the strong interface between components, characterized by van der Waals adhesion and structural interlocking, cracks typically initiate in the matrix between fiber bundles rather than at the fiber‐matrix interface, and they propagate as fiber pull‐out continues.^[^ ^129^ ^]^ However, FRPs only partially replicate native tissues and primarily serve as solutions for achieving high‐strength materials. Extensive efforts are being made in the development of damage‐tolerant materials. For example, Sozumert et al.,^[^ ^130^ ^]^ demonstrated that a nonwoven polypropylene fiber sheet featuring a random fibrous network (without a matrix) exhibited deformation and damage performance similar to that of unnotched samples when subjected to local damage in the form of a longitudinal notch. This finding underscores the critical role of fibrous structures in developing damage‐tolerant materials, as previously demonstrated in SFTs. However, the absence of an aqueous environment limits their functional mimicry.

### 5.2 Hydrogels

Due to their water‐rich composition, hydrogels are suitable for biomedical applications and functionally resemble the GAG and PG matrices in SFTs. These polymers form hydrogen bonds and absorb water, mirroring native soft tissue behavior. These materials possess a unique ability to absorb water and form hydrogen bonds, similar to those in SFTs. However, they are often weak and fragile.^[^ ^131^ ^]^ Hence, incorporating different structural motifs mentioned in Section 2 can significantly improve their function.

### 5.3 Interpenetrating Network and Double Network Hydrogels

Hydrogel modifications—including topological hydrogels,^[^ ^132^ ^]^ nanocomposite hydrogels,^[^ ^133^ ^]^ double‐network (DN) hydrogels,^[^ ^134^ ^]^ and interpenetrating polymer networks (IPNs)^[^ ^131^, ^135^, ^136^ ^]^—have improved toughness and fatigue resistance. Unlike classical hydrogels, which are mechanically weak and not durable, IPNs (where two networks are independently crosslinked—one through covalent bonds and the other through physical bonds) and DNs (where two networks are separately crosslinked, with one consisting of short chains and the other of long chains via covalent bonds) exhibit remarkable toughness and high strain capacity, achieving fracture energies of up to ≈9 kJ m.^−2[^ ^137^ ^]^ Although this behavior is similar to that of native SFTs—where fracture toughness ranges from 1 kJ m^−2^ for porcine aorta to 6 kJ m^−2^ for the temporomandibular joint disc and up to 80 kJ m^−2^ for rhinoceros dermal tissue^[^ ^54^, ^102^ ^]^ (Figure 6)—it is driven by different mechanisms. However, some of these mechanisms are also present in tissues, largely due to the combination of physical and chemical bonds.

In hydrogels, the remarkable fracture toughness arises not only from intrinsic fracture energy dissipation mechanisms like the Mullins effect and bulk hysteretic dissipation (viscoelasticity) but also from localized dissipation near the crack, attributed to the pull‐out of entangled polymer chains and their delocalized damage,^[^ ^136^ ^]^ together with crack pinning and chain sliding.^[^ ^131^ ^]^

Sun et al.^[^ ^137^ ^]^ demonstrated the use of a polyacrylamide (PAAm) and alginate IPN. Upon stretching, the PAAm network provided elasticity and maintained stretchability, while the alginate network dissipated energy by breaking its ionic crosslinks. In pre‐notched samples, the PAAm network acted as a bridge over the crack, while the alginate network unzipped, effectively dissipating energy around the crack tip. This IPN exhibited complete recovery even at 1700% strain, maintaining its structural integrity and achieving a fracture energy of 9 kJ m⁻^2^, even in the presence of a crack.

A primary limitation of IPNs lies in their relatively low stiffness compared to native soft tissues. To address this, composite strategies have been developed to simultaneously enhance toughness and stiffness. Sun et al.^[^ ^138^ ^]^ fabricated a composite hydrogel by embedding rod‐like cellulose nanocrystals (CNCs) within a PAAm matrix, achieving a tenfold increase in crack resistance—up to 1 kJ m^−2^ —relative to PAAm gels alone, without compromising stiffness. Liu et al.,^[^ ^139^ ^]^ introduced an alternative method involving light‐sensitive crosslinkers to induce crack tip softening, resulting in a fourfold enhancement in fracture toughness. Additionally, Zhu et al.^[^ ^140^ ^]^ reported the development of highly entangled DN hydrogels with a combination of high tensile strength (≈3 MPa), fracture energy (8.3 kJ m−2), and a strain‐stiffening capability of 47.5 in 90% water content. These findings underscore the potential of molecular and structural innovations for advancing the mechanical performance of hydrogels.

However, most of these hydrogels rely predominantly on molecular and chemical design strategies, which constrain their mechanical performance to the molecular or nanoscale. Consequently, their strength and stiffness often fall short of those exhibited by native soft fibrous tissues. To achieve concurrent improvements in stiffness, strength, and toughness, it is necessary to integrate additional structural motifs—particularly architectural features spanning multiple length scales, such as those employed in fiber‐reinforced hydrogel composites.

### 5.4 Fiber‐Reinforced Hydrogel Composites and Multiscale Materials

While IPNs provide remarkable fracture toughness and stretchability, they often fall short in replicating the mechanical behavior of SFTs, particularly in stiffness. In contrast, nature achieves these properties through fiber‐reinforced composites. Inspired by this principle, researchers have explored the integration of electrospun nanofibers into hydrogels and IPNs to replicate natural multiscale architectures.^[^ ^141^, ^142^, ^143^, ^144^, ^145^, ^146^, ^147^ ^]^

**Figure 12:**
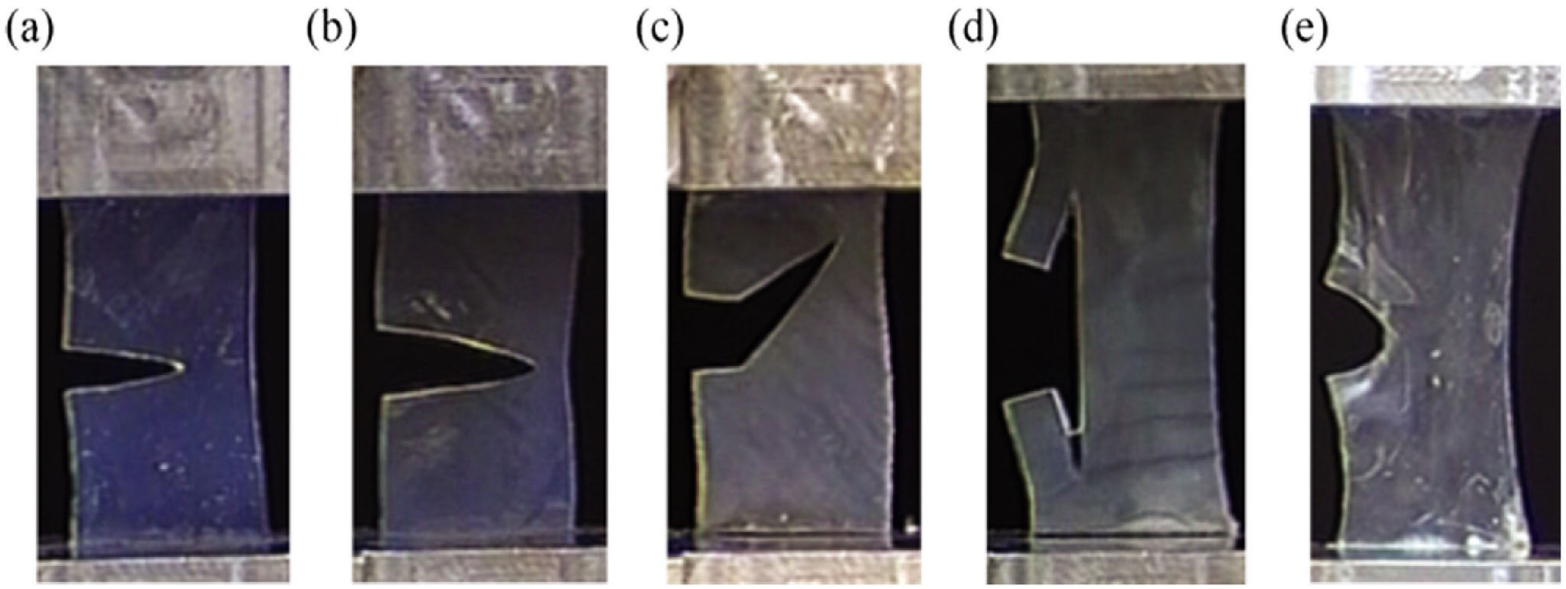
Crack patterns in electrospun gelatin nanofibers reinforced alginate hydrogels. a) no fibers, b) 90° (transverse), c) 45°, d) 0°, and e) cross‐plied (CP) 0°/90°/0°/90°. The orientation is relative to the loading direction. Reproduced under terms of the CC‐BY license^[^ ^141^ ^]^ 2017, Tonsomboon et al., published by Elsevier.

Tonsomboon et al.^[^ ^141^ ^]^ developed biomimetic alginate hydrogels reinforced with electrospun gelatin nanofibers, demonstrating an order‐of‐magnitude increase in strength, with laminating further increasing toughness. The resulting composite exhibited mechanical properties comparable to those of natural soft tissues, and the approach showed adaptability to various fiber‐matrix systems. Furthermore, the study highlighted how fiber orientation influenced crack patterns in the laminates (**Figure** **12**). Notably, stacked laminates were found to blunt crack tips effectively (Figure 12E), showcasing their potential for enhancing durability and fracture resistance in biomimetic materials.

Enhancing hydrogel matrices with nanofibers offers notable advantages, particularly under compressive loading. However, these nanofiber‐reinforced hydrogels often exhibit insufficient bulk stiffness and fail to replicate the characteristic J‐shaped stress–strain response of natural tissues. To overcome these limitations, microfiber‐reinforced hydrogels have been widely explored for biomedical and tissue engineering applications.^[^ ^59^, ^148^, ^149^, ^150^, ^151^, ^152^, ^153^, ^154^, ^155^, ^156^, ^157^ ^]^ Furthermore, integrating hierarchical and multiscale materials into these hydrogels holds significant potential to achieve an optimal combination of stiffness, strength, and toughness akin to that found in SFTs. Guo et al.^[^ ^158^ ^]^ demonstrated the fabrication of a strong and tough hydrogel with architected multiscale hierarchical structures using a freeze‐casting‐assisted solution. This method produced microscale anisotropic honeycomb‐structured fiber walls and matrix, reinforced through dual cross‐linking to enhance hydrogen bonds within fibers as well as nanocrystalline domains and chain‐connecting ionic bonds. The damage tolerance of the material stemmed from the reversible alignment of nanofibers at the crack tip, effectively mimicking the structural and mechanical behaviors observed in SFTs.

**Figure 13:**
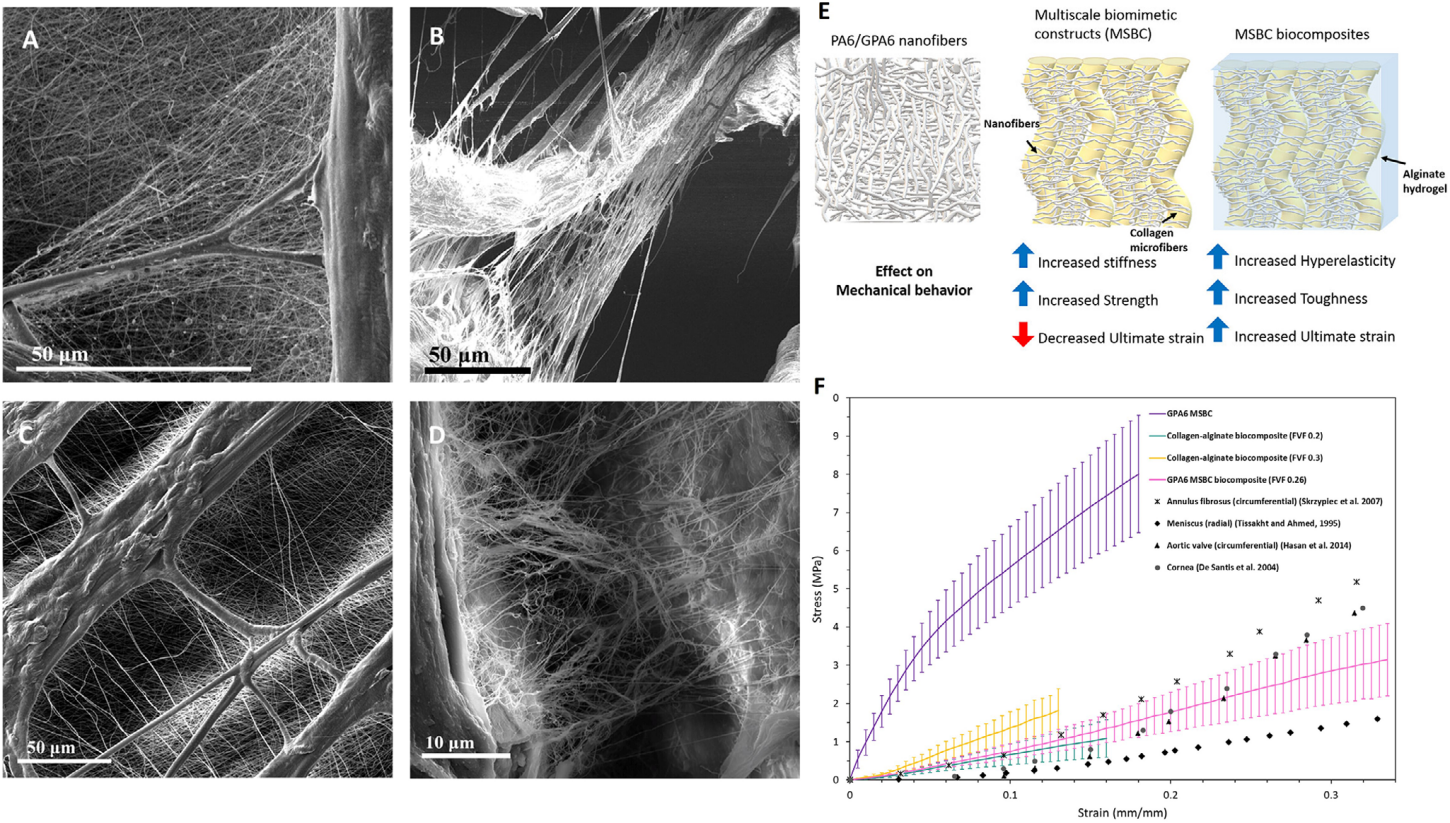
Multiscale biomimetic constructs (MSBCs) (GPA6‐collagen) a,c) compared with the native elastic fibrous network in b,d) an ovine intervertebral disc. Mechanical behavior of multiscale biomimetic constructs (MSBCs) e) illustration of the step‐by‐step influence of fabrication of MSBCs. f) Mechanical behavior of Gelatin‐polyamide 6 (GPA6) ‐collagen MSBC, GPA6‐collagen‐alginate MSBC biocomposites, collagen‐alginate biocomposites, and human SFTs. Mechanical properties of alginate‐collagen biocomposites and MSBC biocomposites. Reproduced under terms of CC‐BY license^[^ ^71^ ^]^ 2023, Sharon et al. published by ACS Publications.

To systematically investigate the mechanical roles of different structural motifs in native tissues, our group developed a series of simplified fiber‐reinforced hydrogel systems. Across multiple studies,^[^ ^71^, ^149^, ^155^, ^156^, ^159^, ^160^, ^161^, ^162^ ^]^ we demonstrated the use of fiber‐reinforced hydrogels to mimic key mechanical attributes of SFTs, including J‐shaped hyperelastic behavior, tensile strength, and stiffness (**Figure** **13**). Additionally, we investigated the role of hierarchical fibers, composed of electrospun nanofibers, in reinforcing the hydrogel matrix and examined the effects of crimping at different scales.^[^ ^159^ ^]^ Interestingly, while nanoscale crimping exerted minimal influence on the toe region of the stress–strain curve, larger‐scale crimping markedly affected the mechanical response—an observation consistent with findings in native tissues.^[^ ^9^ ^]^ More recently, we systematically integrated micro‐ and nanofibers within a hydrogel matrix to evaluate their combined impact on mechanical performance.^[^ ^71^ ^]^ This multiscale design significantly enhanced both toughness and deformation capacity, yielding increases of 140% and 600%, respectively (Figure 13E,F). Notably, comparable multiscale architectures are found in native tissues such as the annulus fibrosus (Figure 13A–D), further reinforcing the translational potential of these biomimetic composites.

### 5.5 Auxetic Materials

Auxetic behavior in metamaterials is typically achieved through a periodic structural arrangement. However, auxetic responses can also emerge from the buckling of random fiber networks via electrospinning. For example, out‐of‐plane buckling of transversely oriented fibers has been shown to produce super‐auxetic behavior, with theoretical Poisson's ratios reaching below −100.^[^ ^163^ ^]^ The fundamental principle behind these super‐auxetic networks is the phenomenon in which certain fibers realign along the direction of the applied force during uniaxial stretching of the fiber network. This reorientation results in lateral contraction of the network. Concurrently, the fibers oriented perpendicular to the force experience compressive stresses, causing them to buckle. The deflected, buckled fibers significantly increase their out‐of‐plane span, leading to a substantial expansion of the overall network.^[^ ^163^ ^]^ Similar buckling behavior is observed in skin tissue, although the effect is less pronounced.^[^ ^51^ ^]^ Lynch et al.^[^ ^51^ ^]^ found that in the heel region of the skin's stress–strain behavior, the proportion of fibers aligned with the applied force exhibits a nonlinear increase as the material is stretched. Conversely, fibers oriented perpendicular to the direction of traction must undergo buckling and bending before they can realign.

Numerous engineered metamaterials have been developed to exhibit auxetic properties.^[^ ^164^, ^165^, ^166^, ^167^, ^168^ ^]^ These materials are gaining prominence in biomedical applications due to their unique capacity to close, rather than open, cracks under tensile loading—a result of their lateral expansion. Such behavior renders them promising candidates for fatigue‐ and tear‐resistant biomaterials, including synthetic skin, vascular grafts, and organ scaffolds.^[^ ^165^, ^167^, ^169^, ^170^, ^171^, ^172^, ^173^ ^]^ One effective strategy for replicating auxetic behavior found in natural systems involves embedding electrospun nanofibers within hydrogels or utilizing the fibers independently.^[^ ^163^, ^174^ ^]^

**Figure 14:**
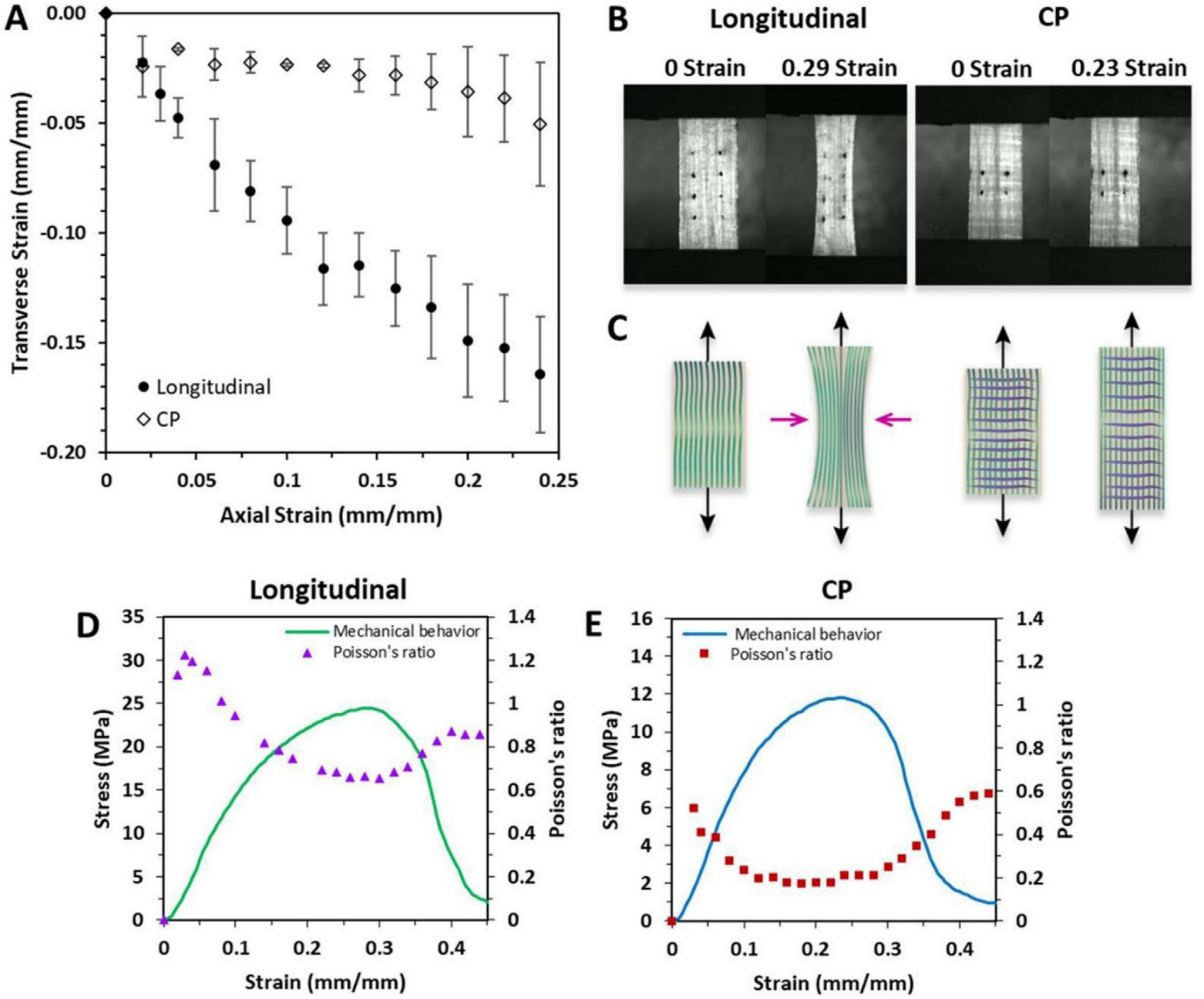
The width changes of the biocomposite laminates. a) The axial versus transverse strains. The cross‐plied (CP) laminates exhibit smaller transverse strains during stretching than the longitudinal laminates. b) The longitudinal and CP laminates at the beginning of stretching (left) and under tension at the UTS point (right). c) Schematic representations of the longitudinal and CP laminates under tension. The center of the longitudinal laminates is significantly narrowed during stretching compared to the CP. The average stress–strain behaviors and Poisson's ratios of the d) longitudinal and e) CP laminates. Reproduced with permission^[^ ^92^ ^]^ 2024, published by Elsevier.

However, SFTs do not frequently exhibit auxetic behavior but instead display non‐standard Poisson's ratios (as discussed in section 2.6), where transverse strain plays a crucial role in closing tears and cracks. We observed similar phenomena in our fibroin‐alginate biocomposites, wherein structural shielding was demonstrated by comparing longitudinal and cross‐plied configurations^[^ ^92^, ^156^ ^]^ (**Figure** **14**).

### 5.6 Mechanical Biocompatibility in Biomimetic Materials

**Figure 15:**
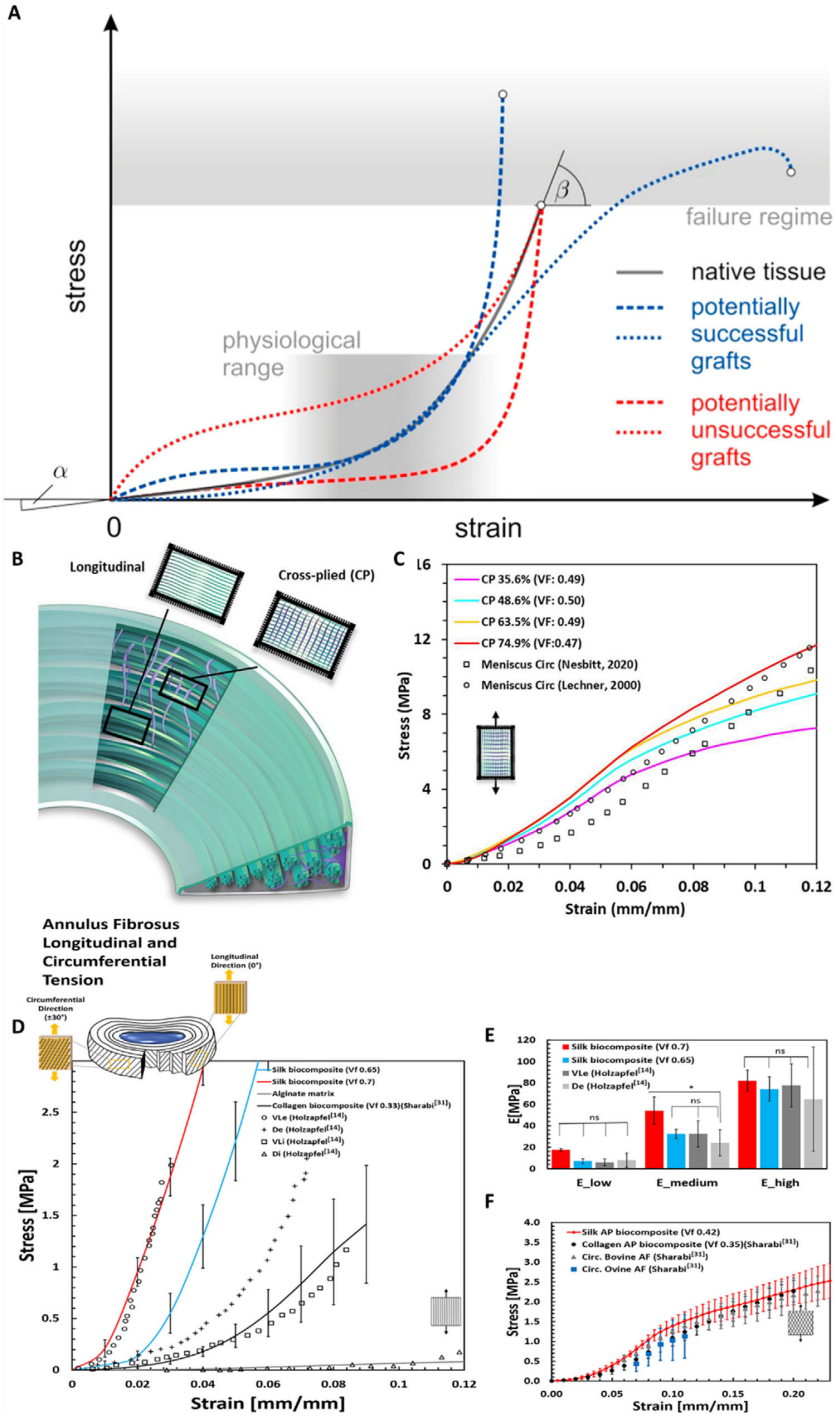
Mechanical biocompatibility of biomedical materials. a) Schematic stress–strain curves comparing native tissue and potential grafts. Despite matching certain mechanical properties, grafts may fail to replicate the native tissue response within the physiological loading range. Reproduced with permission^[^ ^5^ ^]^ 2015, Mazza & Ehret. Published by Elsevier. b,c) Mechanical biocompatibility of the meniscus. Schematic representation of the circumferential and radial fibers in the meniscus and the mechanical behavior of silk fiber‐reinforced hydrogel composite with the human meniscus tissue. Reproduced with permission^[^ ^92^ ^]^2024, Aharonov et al. Published by Elsevier. D–F) Mechanical biocompatibility of the annulus fibrosus (AF). Mechanical behavior of fiber‐reinforced biocomposites in longitudinal and angle‐plied (±30°) orientations compared with human AF in the longitudinal (d,e) and circumferential (f) directions, respectively. Reproduced under the terms of the CC‐BY‐NC‐ND with permission^[^ ^155^ ^]^ 2023, Mordechai et al. Published by Wiley.

*Mechanical biocompatibility* refers to the capacity of a material to replicate the complete mechanical behavior of a specific tissue, as defined by Mazza and Ehret (2015).^[^ ^5^ ^]^ For soft, highly deformable tissues such as SFTs, achieving mechanical biocompatibility requires more than matching isolated properties (e.g., tensile strength or modulus); it necessitates emulating the full deformation response (**Figure** **15**A). This remains a significant challenge due to the complex, anisotropic, nonlinear, and viscoelastic nature of SFTs.

Comparative mechanical characterization between biomimetic materials and native tissues must account for multiple variables—including experimental protocol, length scale, and loading orientation—all of which profoundly affect the observed behavior. The absence of standardized testing procedures further complicates such assessments.

Nonetheless, the systematic incorporation of native structural motifs into biomimetic materials offers a promising pathway toward achieving mechanical biocompatibility. We have demonstrated this approach in both meniscal ^[^ ^92^ ^]^ and annulus fibrosus tissues^[^ ^155^ ^]^ comparing the behavior of bioinspired composite laminates with that of native human tissues, wherever feasible ^[^ ^47^, ^175^, ^176^ ^]^ (Figure 15B–F). Critically, these comparisons must be conducted under consistent experimental conditions, such as equivalent loading rates, orientations, preconditioning protocols, and hydration states, to yield meaningful insights into mechanical biocompatibility.

## 6 Conclusions and Closing Remarks

Designing material systems inspired by nature yet enhanced by advanced technologies holds immense potential for addressing critical challenges in biomaterials research.^[^ ^177^ ^]^ Yet, many synthetic materials still lack the mechanical biocompatibility required to integrate seamlessly with native soft tissues. Despite growing interest and the availability of diverse biomaterials and applications, general guidelines remain lacking for systematically studying SFTs and translating their structure–mechanics relationships into engineered designs. This underscores the need for hierarchical, motif‐based architectures that replicate not only the biological composition but also the mechanical performance of native tissues.

Employing simple, repeating principles to achieve diverse and extraordinary behaviors offers a strategic framework for creating synthetic materials that can effectively substitute for native tissues. While biological compatibility is widely recognized, mechanical biocompatibility—required to match the J‐shaped hyperelastic response, high deformability, and failure tolerance of SFTs—remains underexplored and underdeveloped in current materials. This limitation continues to hinder progress in tissue repair, biomedical engineering, regenerative medicine, and soft robotics.

Integrating generative artificial intelligence (AI) tools—such as large language models (LLMs) and multi‐agent systems—offers transformative potential for accelerating the discovery and design of biomimetic materials.^[^ ^178^, ^179^, ^180^, ^181^ ^]^ When coupled with advanced fabrication technologies and multiscale analysis of structure–mechanics relationships, these tools can redefine the biomimetic design process. AI enhances creativity, efficiency, and predictive capability, enabling data‐driven optimization of material architectures.

Ultimately, the future of biomimetic materials lies in engineering synthetic systems that unite hyperelasticity, strength, flaw insensitivity, high deformation capacity, and fatigue resistance. By emulating the structural motifs of SFTs and capturing their intrinsic structure–function logic, we can create next‐generation materials that are both biologically and mechanically compatible—capable of meeting the most demanding challenges in healthcare and beyond.

## Conflict of Interest

The authors declare no conflict of interest.
